# Neurons with granulovacuolar degeneration bodies are resilient to tau-induced protein synthesis impairment

**DOI:** 10.1126/sciadv.aea8940

**Published:** 2026-03-06

**Authors:** Jasper F. M. Smits, Thijmen W. Ligthart, Marta Jorge-Oliva, Skip Middelhoff, Fleur Schipper, Débora Pita-Illobre, Ka Wan Li, Wiep Scheper

**Affiliations:** ^1^ Dept. of Human Genetics, Amsterdam UMC - Vrije Universiteit Amsterdam, Amsterdam, The Netherlands.; ^2^ Dept. of Functional Genomics, Vrije Universiteit Amsterdam, Amsterdam, The Netherlands.; ^3^ Dept. of Molecular and Cellular Neuroscience, Center for Neurogenomics and Cognitive Research, Amsterdam Neuroscience, Vrije Universiteit Amsterdam, Amsterdam, The Netherlands.

## Abstract

In Alzheimer’s disease, many surviving neurons with tau pathology contain granulovacuolar degeneration bodies (GVBs), neuron-specific lysosomal structures induced by pathological tau assemblies. This could indicate a neuroprotective role for GVBs; however, the mechanism of GVB formation and its functional implications are elusive. Here, we demonstrate that casein kinase 1δ (CK1δ) activity is required for GVB formation. CK1δ is sequestered in the GVB during this process in an autophagy-dependent manner. We show that neurons with GVBs (GVB^+^) are resilient to tau-induced impairment of global protein synthesis and are protected against tau-mediated neurodegeneration. GVB^+^ neurons do not exhibit differential activation of transient translational stress responses but have increased ribosomal content. Unlike neurons without GVBs, GVB^+^ neurons fully retain the capacity to induce long-term potentiation–induced protein synthesis in the presence of tau pathology. Our results have identified CK1δ as a key regulator of GVB formation that confers a protective neuron-specific stress response to tau pathology. These findings provide opportunities for targeting neuronal resilience in tauopathies.

## Teaser

GVB+ neurons resist tau toxicity and maintain protein synthesis rates, revealing a protective, neuron-specific response.

## INTRODUCTION

The progressive intraneuronal accumulation of aggregated proteins is central in the pathogenesis of neurodegenerative diseases, with tau protein in Alzheimer’s disease (AD) and frontotemporal dementias (FTDs) as a prime example (*1*). Neurons depend on robust protein homeostasis (proteostasis) to acquire and maintain properties required for neuronal function. Moreover, postmitotic neurons cannot self-renew, and their unique features that are acquired throughout their lifetime make them difficult to replace. Pathological tau accumulation impairs neuronal protein synthesis (*2*–*6*), folding (*7*) and degradation (*8*–*10*), thereby strongly challenging proteostasis. Consequently, neurons must have intrinsic mechanisms to withstand proteostatic disturbances. Granulovacuolar degeneration bodies (GVBs) are membrane-delineated structures that accumulate in the soma of a subset of neurons affected by early stages of tau pathology (*11*–*13*). GVBs are associated with tau pathology not only in AD and FTD but also in other tauopathies like progressive supranuclear palsy (*14*), Pick’s disease (*15*), as well as Down syndrome (*16*, *17*) [reviewed before (*13*, *18*)], yet their functional role in tau pathogenesis remains unclear. The high number of GVB^+^ neurons in brains of cognitively healthy centenarians (*19*, *20*) and the observation that most surviving neurons in the AD hippocampus have GVBs (*21*, *22*) suggest that the presence of GVBs may signify a protective proteostatic response. It has been hypothesized that GVBs may protect neurons by sequestering damaging proteins (*13*, *18*, *21*). Alternatively, the direct connection with pathological tau accumulation could suggest that GVBs function to enhance tau degradation to restore proteostasis. However, there is no convincing evidence for the accumulation of tau in GVBs (*18*, *23*), raising the possibility that the presence of GVBs affects neurons via an alternative mechanism. Here, we investigated the formation and functional significance of GVBs to elucidate a previously unknown molecular mechanism underlying neuronal resilience to pathological tau-induced proteostatic disturbance.

GVBs were merely enigmatic appearances in postmortem brain tissue until our laboratory recently developed experimental models of tau-induced GVB formation that allow mechanistic study (*23*–*25*). Tau aggregates in these models are detergent-insoluble fibrillar structures with high β sheet content, which are positive for multiple pathology-associated phospho-specific tau antibodies (*25*, *26*). We have demonstrated that the formation of GVBs is a neuron-specific response that is triggered by intraneuronal pathological protein assemblies (*23*, *24*). GVBs are proteolytically active lysosomal structures that contain endo- and autosomal cargo accumulated in one or multiple dense cores (*23*). These cores are strongly immunopositive for several epitopes, including phosphorylated protein kinase R–like endoplasmic reticulum kinase (pPERK) (*18*) that are useful as markers to detect GVBs. However, it is largely unknown whether the immunopositivity of the GVB core reflects the presence of specific proteins or just protein fragments or neo-epitopes [extensively reviewed before (*13*, *18*)]. To date, casein kinase 1δ (CK1δ) is the only protein validated to selectively accumulate in GVBs using direct fluorescence (*23*). CK1δ is a key GVB marker that consistently localizes to GVBs in both in vivo mouse models (*23*, *27*–*32*) and human postmortem brain tissue (*13*, *18*). CK1δ belongs to the casein kinase family, which comprises seven members (α, β, γ1–3, δ, and ε), and is an evolutionarily conserved serine/threonine kinase that is ubiquitously expressed and has pleiotropic functions (*33*, *34*). Notably, CK1δ and its yeast ortholog Hrr25 have been implicated in several fundamental pathways central to proteostasis, including ribosome assembly (*35*–*37*), endocytosis (*38*, *39*), and autophagy (*40*–*43*). However, the mechanism of the formation of GVBs and the potential functional involvement of CK1δ therein is completely unknown.

In the present work, we demonstrate that neurons with GVBs (GVB^+^ neurons) are resilient to pathological tau-mediated neurodegeneration. We identified CK1δ activity as a rate-limiting factor in the formation of GVBs and showed that CK1δ sequestration in the GVB requires the autophagic machinery. Neurons with GVBs have an increase in ribosomal content and are resilient to tau-induced impairment of global protein synthesis. GVB^+^ neurons are resilient to the associated impairment of long-term potentiation (LTP)–regulated protein synthesis. Our results demonstrate that GVB^+^ neurons are protected against protein synthesis impairment and neurodegeneration induced by tau pathology.

## RESULTS

### GVB^+^ neurons are resilient to tau-induced neurodegeneration

**Fig. 1.**
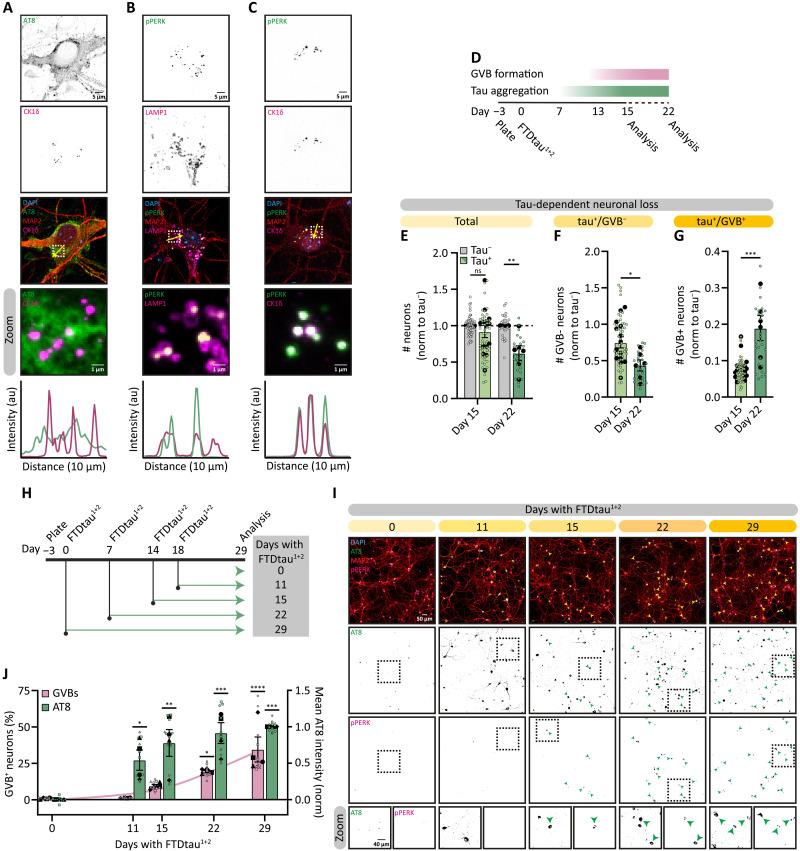
GVB^+^ neurons are resilient to tau-induced neurodegeneration. (**A** to **C**) Representative confocal images of tau^+^/GVB^+^ neurons at day 22 showing AT8 (green) and CK1δ (magenta) (A), pPERK (green) and CK1δ (magenta) (B), pPERK (green) and LAMP1 (magenta), (C) and MAP2 (red). Dashed squares indicate zoomed regions, yellow arrows indicate the location of the intensity profiles. (**D**) Experimental timeline. (**E** to **G**) Tau-dependent neuronal loss in the total tau^+^ population (E), tau^+^/GVB^−^ (F) or tau^+^/GVB^+^ (G) neurons at day 15 or day 22, normalized to the number of tau^−^ neurons (*N* = 6 to 14; *n* = 27 to 54). For visual clarity, biological replicates are not distinguished in shape. (**H**) Protocol used to assess progression of tau aggregation and GVB^+^ formation by transduction with FTDtau^1+2^ at the indicated points. (**I**) Representative high-content microscopy images at different FTDtau^1+2^ exposure durations as indicated. Shown are AT8 (green), MAP2 (red), and pPERK (magenta). Dashed squares indicate zoomed regions. Arrowheads indicate GVB^+^ neurons. (**J**) Percentage of GVB^+^/tau^+^ neurons and mean AT8 intensity normalized to 29 days FTDtau^1+2^ (1) and untransduced (0) (*N* = 4; *n* = 10 TO 12). Nuclei are in blue, separate channels are in grayscale [(A) to (C) and (I)]. Data are presented as means ± SEM. Nested *t* test (E to G) and nested one-way analysis of variance (ANOVA) followed by Dunnett’s post hoc test (J) were used. *P < 0.05, **P < 0.01, ***P < 0.001, ****P < 0.0001; ns, not significant. au, arbitrary units. Details of replicates and number of neurons analyzed in tables S1 and S2.

To study the formation of GVBs in relation to tau pathology over time, we used our extensively characterized and validated primary neuron model for seed-independent tau pathology where lentiviral transduction with FTDtau^1 + 2^ (2N4R human tau with the P301L/S320F FTD mutations) results in progressive pathological tau accumulation accompanied by GVB formation (Fig. 1A) (*24*, *25*). Like GVBs observed in the human brain, these GVBs contained a core, positive for CK1δ and pPERK (Fig. 1B), delineated by a lysosomal membrane (Fig. 1C) as previously shown (*24*). Fifteen days after FTDtau^1+2^ transduction, around 10% of the neurons with pathological tau assemblies have formed GVBs (*24*, *25*) (tau^+^/GVB^+^ neurons). At this point, neuronal loss or degeneration of neurites or synapses was not observed (fig. S1, A to G), and calcium imaging analysis showed that neuronal network activity was unaffected (fig. S1, H to L), indicating that this time point represents a stage in tau pathogenesis that precedes neurodegeneration. However, exposure to tau pathology for an additional week (Fig. 1D) induced neuronal loss (Fig. 1E). In addition, analysis at 15 days using different FTDtau^1+2^ concentrations showed that there is a strong correlation between the extent of tau pathology and neuronal loss (fig. S1M). In line with studies in human brain (*21*), this demonstrated that in contrast to the loss of GVB-negative (GVB^−^) neurons as tau pathology progresses (Fig. 1F), the fraction of GVB^+^ neurons increased (Fig. 1G), indicating that GVB^+^ neurons are more resilient to tau-induced neurodegeneration. To study the temporal dynamics of GVB formation, the percentage of GVB^+^ neurons was determined at different incubation periods with FTDtau^1+2^ (Fig. 1, H and I). While the accumulation of MC1^−^ and AT8-positive tau assemblies started to plateau after 22 days of exposure to FTDtau^1+2^, the fraction of GVB^+^ neurons continued to increase up to 34% after 29 days (Fig. 1, I and J, and fig. S1, M and N). Together, these findings highlight that GVB formation progresses whereas GVB^−^ neurons are lost to tau aggregation, suggesting that GVBs play a role in neuronal resilience to tau-induced neurodegeneration.

### CK1δ activity is a key regulator of GVB formation

**Fig. 2.**
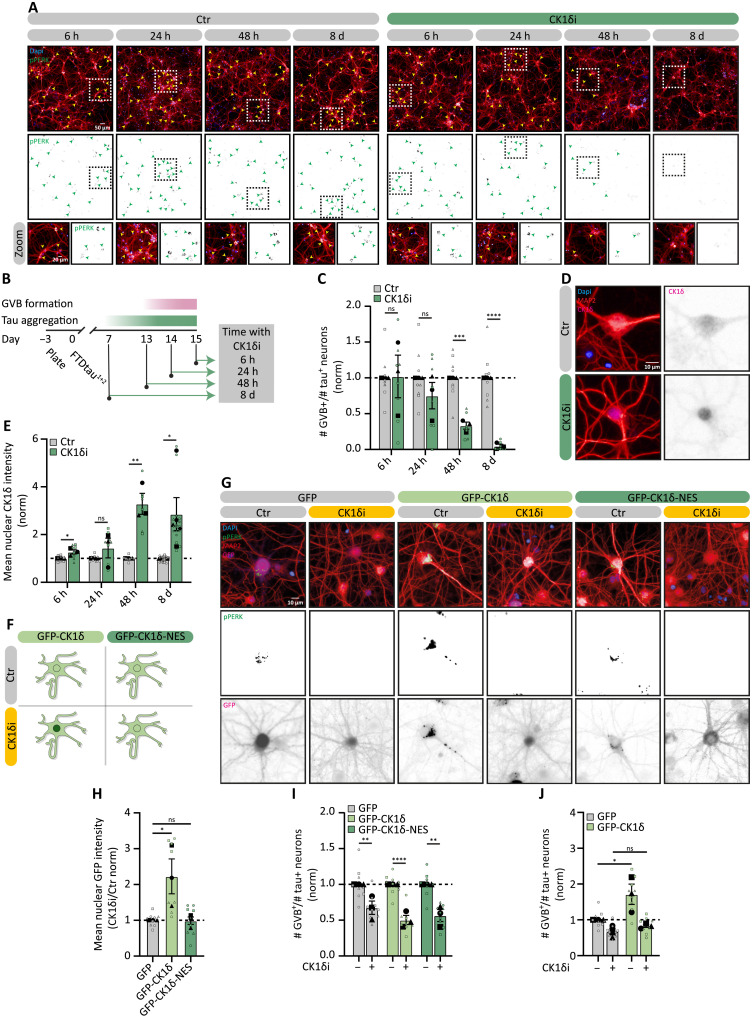
CK1δ is a key regulator for the formation of GVBs. (**A**) Representative high-content microscopy images of tau^+^ neurons w/wo CK1δ_i_ treatment for 6, 24, and 48 hours (h) or 8 days (d) analyzed at day 15. Shown are MAP2 (red) and pPERK (green). Arrowheads indicate GVB^+^ neurons; dashed squares indicate zoomed regions. (**B**) Experimental timeline. (**C**) Fraction of GVB^+^/tau^+^ (pPERK based) neurons with CK1δ_i_ normalized to untreated per time point (*N* = 3; *n* = 7 to 8). (**D**) Representative high-content microscopy images of tau^+^ neurons at day 15 w/wo CK1δ_i_ for 48 hours, shown are MAP2 (red) and CK1δ (magenta). (**E**) Mean nuclear CK1δ intensity of tau^+^ neurons at day 15 with CK1δ_i_ normalized to untreated per time point (*N* = 3 to 5; *n* = 8 to 17). (**F**) Schematic of GFP-CK1δ and GFP-CK1δ-NES localization w/wo CK1δ_i_. (**G** to **I**) Tau^+^ neurons transduced with GFP, GFP-CK1δ, or GFP-CK1δ-NES treated w/wo CK1δ_i_ for 48 hours and analyzed at day 22. (G) Representative high-content microscopy images showing GFP (magenta), MAP2 (red), and pPERK (green). (H) Mean nuclear GFP intensity upon CK1δ_i_, normalized per group and subsequently to the response in GFP-transduced neurons (*N* = 3; *n* = 8-9). (I) Fraction of GFP^+^ GVB^+^/tau^+^ (pPERK based) neurons normalized to untreated per group (*N* = 3; *n* = 8 to 9). (**J**) Fraction of GFP^+^ GVB^+^/tau^+^ (pPERK based) neurons transduced with GFP or GFP-CK1δ treated w/wo CK1δ_i_ for 48 hours, analyzed at day 22, and normalized to untreated GFP-transduced neurons (*N* = 3; *n* = 8 to 9). Nuclei are in blue; separate channels are in grayscale [(A), (D), and (G)]. Data are presented as means ± SEM. Nested t-test [(C), (E), and (I)], nested one-way ANOVA followed by Dunnett’s post hoc test (H) or Sidak’s post hoc test (J) were used. *P < 0.05, **P < 0.01, ***P < 0.001, ****P < 0.0001; ns, not significant. Details of replicates and number of neurons analyzed in tables S1 and S2.

To investigate the molecular mechanism associated with the formation of GVBs, the kinase CK1δ is a prime candidate because it selectively accumulates in GVBs (*23*). To study the role of CK1δ, tau^+^ neurons were treated with the CK1δ/ε-specific ATP-competitive kinase inhibitor PF670462 (CK1δ_i_) (*44*, *45*). We studied the effect of CK1δ_i_ 15 days after FTDtau^1+2^ transduction. CK1δ_i_ treatment was initiated either before or after the first GVBs are detected at day 13 (Fig. 2, A and B) (*24*). CK1δ_i_ did not induce toxicity or negatively affect neuronal morphology or synapse number (fig. S2, A to M). Using pPERK as a GVB marker, we found that CK1δ_i_ treatment for 48 hours or 8 days reduced the number of GVB^+^ neurons to 33 and 4% of control, respectively (Fig. 2, A and C). This shows that the formation of GVBs is reduced by CK1δ_i_ when initiated before or during the first GVBs occur. In contrast, CK1δ_i_ treatment for 24 hours or less did not significantly change the number of GVB^+^ neurons (Fig. 2, A and C). To test whether this was due to the shorter incubation period or whether CK1δ_i_ has no effect once GVB formation is in progress, tau pathology exposure was extended to 22 days, with treatment starting well after the onset of GVB formation (fig. S2N). The number of GVB^+^ neurons was also strongly reduced upon CK1δ_i_ treatment after 48 hours (63% of control) and 8 days (12% of control) (fig. S2O), indicating that CK1δ is required both for the initiation and progression of GVB formation. To exclude that the effect of CK1δ_i_ was due to altered phosphorylation of GVB-cargo that may affect the detection of phospho-epitopes like pPERK, we tested the effect of CK1δ_i_ using a non–phospho GVB marker that has been identified in AD postmortem brain, GOLGINA4 (*32*, *46*). GOLGINA4 was validated as a bona fide GVB marker in our experimental model as well (fig. S3, A and B). CK1δ_i_ treatment strongly reduced GOLGINA4-detected GVB^+^ neurons to 27% of control (fig. S3C), confirming that CK1δ_i_ decreases GVB formation. CK1δ is reported to phosphorylate tau in vitro (*47*, *48*); therefore, we assessed pathological tau accumulation as previously described (*24*–*26*). Treatment with CK1δ_i_ for 48 hours or 8 days did not affect the level of MC1-positive FTDtau^1+2^ accumulation (fig. S3, D and F) and the phospho-epitope–independent direct fluorescence intensity of MeOH-insoluble FTDtau^1+2^–green fluorescent protein (GFP) (fig. S3, E and G). This indicates that the effect of inhibition of CK1δ activity on the number of GVB^+^ neurons is not caused by an indirect effect on tau pathology. Together, these data collectively place CK1δ upstream in the pathway driving GVB formation.

To further confirm the key role of CK1δ in GVB generation, short hairpin RNA (shRNA)–mediated knock-down (KD) was used to reduce CK1δ protein levels (fig. S4, A to C). *shCsnk1d* significantly reduced the fraction of GVB^+^ neurons to 52% of control (fig. S4, D and E). Because of the high toxicity associated with *shCsnk1d* treatment (fig. S4F), overexpression of an *shRNA*-resistant CK1δ variant (GFP-CK1δ_RES_) was used to distinguish the effects of CK1δ KD on the formation of GVBs from its toxic effects (fig. S4, G to J). This was sufficient to rescue the toxic effect of CK1δ KD (fig. S4K). Moreover, overexpression of GFP-CK1δ_RES_ rescued the inhibition of GVB accumulation induced by *shCsnk1d* (fig. S4L), further supporting an essential role for CK1δ in the formation of GVBs.

Next, we investigated whether regulation of CK1δ levels or activity underlies its role in GVB formation. Tau aggregation did not alter the total protein level of CK1δ (fig. S5, A and B) with no difference in somatic CK1δ intensity between GVB^−^ and GVB^+^ neurons (fig. S5C). In contrast, treatment with CK1δ_i_ strongly increased CK1δ levels, independent of the presence of tau (fig. S5, A and B). Moreover, CK1δ_i_ increased nuclear CK1δ levels (Fig. 2, D and E, and fig. S2P), in line with a previous study that showed localization of inactive CK1δ to the nucleus (*49*). To exclude that the effect of CK1δ_i_ on GVBs was indirectly caused by a changed balance in cytosolic-to-nuclear CK1δ levels, we used GFP-CK1δ (*23*) with a nuclear export signal (GFP-CK1δ-NES) to force its cytosolic localization (Fig. 2F). GFP-CK1δ-NES selectively localized to GVBs (fig. S5, D and E), indicating that the NES sequence does not interfere with the localization of CK1δ to GVBs. Upon treatment with CK1δ_i_, GFP-CK1δ relocalized to the nucleus like endogenous CK1δ, whereas—as expected—the localization of GFP-CK1δ-NES was not changed (Fig. 2, G and H). However, neither GFP-CK1δ nor GFP-CK1δ-NES was able to rescue the CK1δ_i_-induced decrease in number of GVB^+^ neurons (Fig. 2I), indicating that the activity of CK1δ directly affects the formation of GVBs. To further support the direct involvement of CK1δ activity in GVB formation, we compared the proportion of GVB^+^ neurons following CK1δ overexpression with those overexpressing GFP alone in tau^+^ neurons (Fig. 2G). GFP-CK1δ^+^ neurons were 1.72 times more likely to form GVBs than GFP^+^ neurons (Fig. 2J). Similarly, overexpression of the cytosolic variant GFP-CK1δ-NES also enhanced GVB formation in tau^+^ neurons (2.36-fold relative to GFP; fig. S5F). The effect of both GFP-CK1δ and GFP-CK1δ-NES overexpression on GVBs was lost when neurons were treated with CK1δi (Fig. 2J and fig. S5F). Together, these results demonstrate that the activity of cytosolic CK1δ is required to generate GVB^+^ neurons.

### CK1δ accumulation in GVBs marks the appearance of GVB^+^ neurons

**Fig. 3.**
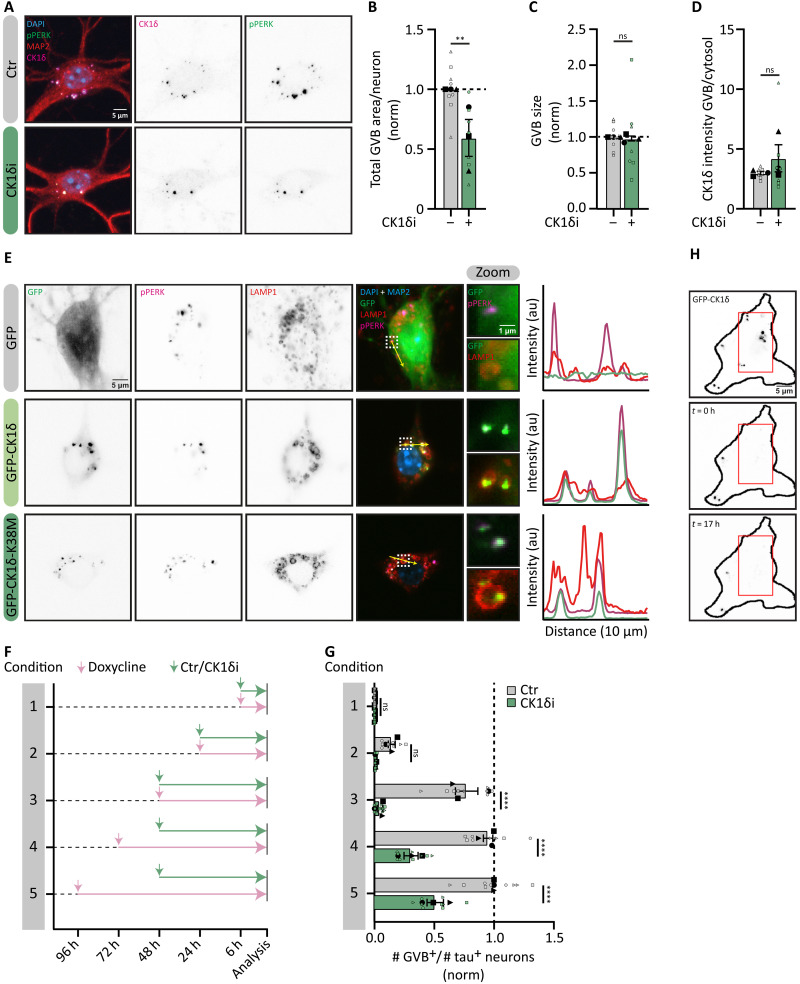
CK1δ accumulation in GVBs marks the appearance of GVBs. (**A** to **D**) Effect of CK1δ_i_ 48 hours of treatment on tau^+^/GVB^+^ neurons. (A) Representative confocal images. Maximum intensity projected Z-stacks showing CK1δ (magenta), pPERK (green), and MAP2 (red). (B to D) High-content quantification of pPERK^+^ GVB-area per neuron (B), single GVB size (C), and the GVB/cytosol ratio of CK1δ intensity (D) at day 15. Data were normalized to control (B and C) (*N* = 3; *n* = 7 to 8). (**E**) Representative confocal images of tau^+^/GVB^+^ neurons transduced with GFP, GFP-CK1δ, or GFP-CK1δ-K38M analyzed at day 22. Shown are GFP (green), pPERK (magenta), LAMP1 (red), and MAP2 (blue). Dashed squares indicate zoomed regions; yellow arrows indicate the location of the intensity profiles. (**F** and **G**) Tau^+^ neurons were transduced with Dox-GFP-CK1δ, treated with Dox and w/wo CK1δ_i_ for different durations as indicated. (F) Experimental timeline. (G) Fraction of GFP^+^ GVB^+^/tau^+^ (Dox-GFP-CK1δ based) neurons at day 22 after Dox treatment w/wo CK1δ_i_ as specified in (F), normalized to neurons treated for 96 hours with Dox wo CK1δ_i_ analyzed (*N* = 3; *n* = 3 to 8). (**H**) Representative live confocal images of tau^+^/GVB^+^ neurons transduced with GFP-CK1δ and followed over time after FRAP at day 22. Shown are cells directly after FRAP (*t* = 0 hours) and at the end of the experiment (*t* = 17 hours). The red square indicates the region that was exposed to FRAP. Nuclei in blue, separate channels are in grayscale [(A) and (E)]. Data are presented as means ± SEM. Nested *t* test [(B) to (D)] and nested one-way ANOVA followed by Sidak’s post hoc test (G) were used. **P < 0.01, ****P < 0.0001; ns, not significant. au, arbitrary units. Details of replicates and number of neurons analyzed in tables S1 and S2. h, hours.

To better understand the connection between the role of CK1δ activity in GVB formation and its accumulation inside GVBs, we first investigated whether the effect of CK1δ_i_ on GVB^+^ neurons is binary (GVB^+^ or GVB^−^ neuron) or more continuous (affecting the number of GVBs per neuron). Using high-resolution confocal microscopy, reduced GVB content per neuron upon CK1δ_i_ treatment was observed (Fig. 3A). Quantification by high-throughput microscopy confirmed that inhibition of CK1δ decreased the total area of GVBs within a single neuron but not the size or the GVB over cytosol ratio of CK1δ fluorescence intensity of individual GVBs (Fig. 3, B to D). These data indicate that CK1δ activity stimulates the formation of GVBs also after the first GVBs have formed. To investigate the role of CK1δ activity in CK1δ localization to GVBs more directly, a kinase-dead K38M CK1δ mutant was used (fig. S5E) (*49*, *50*). GFP-CK1δ-K38M selectively accumulates in GVBs (Fig. 3E). This indicates that although CK1δ activity is necessary for GVB formation, the activity of an individual CK1δ molecule (in cis activity) is not required for its specific accumulation in the core of GVBs.

To study the dynamics of CK1δ accumulation in GVBs, we used a doxycycline (Dox)–inducible expression system for GFP-CK1δ (Dox-GFP-CK1δ; fig. S6, A and B). Dox-GFP-CK1δ was first detected in GVBs 24 hours after induction and strongly increased at 48 hours, after which it started to plateau (Fig. 3, F and G), indicating that a steady state of newly labeled GVBs is obtained at this point. In the presence of CK1δ_i_ (conditions 1 to 3; Fig. 3F), the formation of neurons with Dox-GFP-CK1δ^+^ GVBs was completely prevented (Fig. 3G). Because CK1δ activity in cis is not required for GVB localization (Fig. 3E), this suggests that the targeting of CK1δ to the core of GVBs is directly coupled to the formation of GVBs. To formally exclude the possibility that rather than inhibiting the formation of new GVBs, CK1δ_i_ stimulates the turnover of already formed GVBs, Dox-GFP-CK1δ expression was induced 24 or 48 hours before CK1δ_i_ treatment (condition 4 and 5; Fig. 3F). CK1δ_i_ treatment reduced the number of Dox-GFP-CK1δ^+^/GVB^+^ neurons but did not completely block it (Fig. 3G). This indicates that the remaining Dox-GFP-CK1δ^+^/GVB^+^ neurons were formed before CK1δ_i_ was added. Consequently, we were able to calculate the stability of GVBs. Since CK1δi blocks new GVB formation, the observed GVB^+^ neurons (fig. S2O) existed before treatment. The difference between expected (Fig. 1H) and observed GVB^+^ neurons (fig. S2O) was used to calculate an average *t_1/2_* of 5.4 days (fig. S6C). This is supported by live cell microscopy of GFP-CK1δ–labeled GVBs, demonstrating that GVBs were relatively stable for at least 9 hours (movie S1), indicating a low-turnover rate. Furthermore, using fluorescence recovery after photobleaching (FRAP) imaging, we observed that no new CK1δ is incorporated into the preexisting GVBs over a span of ±17 hours (Fig. 3H). These data collectively identify CK1δ as an upstream regulator of the pathway that makes neurons GVB-positive and indicate that CK1δ is targeted to the GVB core during this process. Given the stability of the CK1δ accumulations in GVBs, they reflect the initiation of the GVB condition in the neuron.

### Cargo accumulation in GVBs requires autophagy

**Fig. 4.**
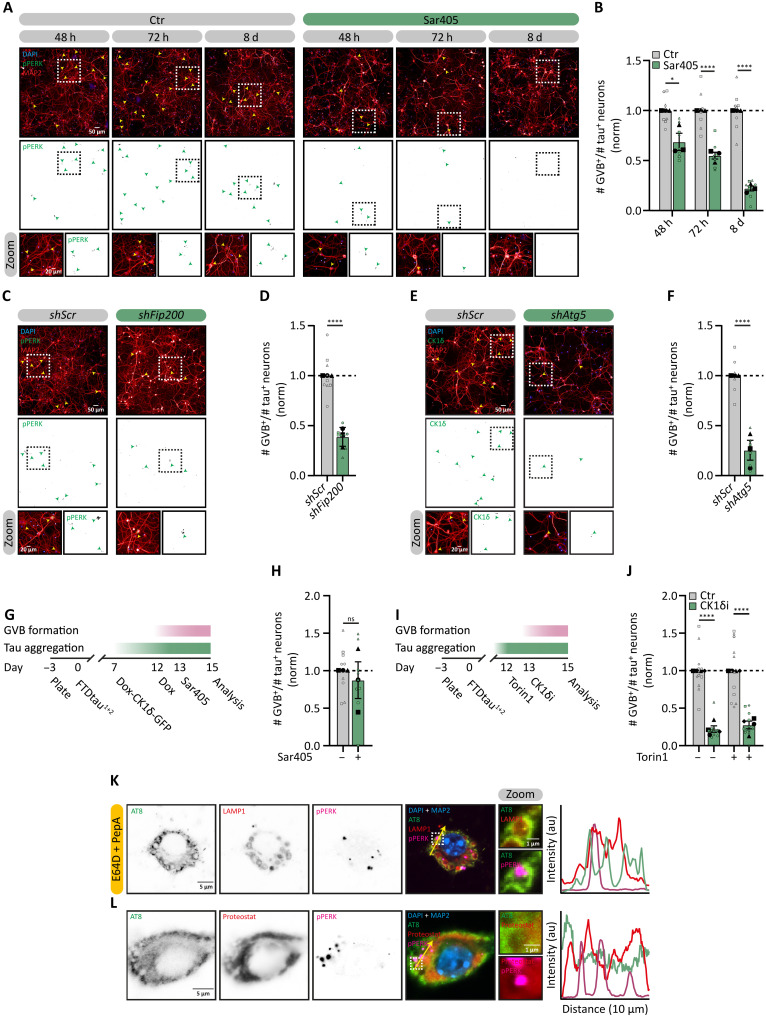
CK1δ sequestration into the GVB requires basal autophagy. (**A** and **B**) Tau^+^ neurons were treated w/wo Sar405 as indicated and analyzed at day 15. (A) Representative high-content microscopy images showing pPERK (green) and MAP2 (red). (B) Fraction of GVB^+^/tau^+^ (pPERK based) neurons upon Sar405 treatment normalized to untreated per time point (*N* = 3; *n* = 8 to 9). (**C** to **F**) Tau^+^ neurons were transduced with *shScr*, *shFip20*0 (C and D), or *shAtg5* (E and F) 8 days before analysis at day 15. (C and E) Representative high-content microscopy images showing MAP2 (red) and pPERK (C) or CK1δ (E) (green). (D and F) Fraction of GVB^+^/tau^+^ (pPERK (D) or CK1δ (F) based) neurons with *shFip200* (D) or *shAtg5* (F) normalized to *shScr* (*N* = 3; *n* = 6 to 9). (**G** and **H**) Neurons transduced with Dox-GFP-CK1δ were treated with Dox for 72 hours and w/wo 48 hours of Sar405 analyzed at day 15. (G) Experimental timeline. (H) Fraction of GFP^+^ GVB^+^/tau^+^ (Dox-GFP-CK1δ based) neurons, normalized to untreated (*N* = 3; *n* = 9). (**I** and **J**) Tau^+^ neurons were treated with Torin1 and subsequently w/wo CK1δi analyzed at day 15. (I) Experimental timeline. (J) Fraction GVB^+^/tau^+^ (pPERK based) neurons normalized to untreated (*N* = 4; *n* = 11 to 12). (**K**) Representative confocal images of tau^+^/GVB^+^ neurons treated with E64D and pepstatin A (PepA) for 24 hours analyzed at day 15. Shown are AT8 (green), LAMP1 (red), pPERK (magenta), and MAP2 (blue). (**L**) Representative confocal images of tau^+^/GVB^+^ neurons at day 15 showing Proteostat (red), AT8 (green), pPERK (magenta), and MAP2 (blue). Nuclei are in blue, separate channels in grayscale, dashed squares indicate zoomed regions [(A), (C), (E), (K), and (L)], arrowheads indicate GVB^+^ neurons [(A), (C), and (E)], and yellow arrows indicate the location of the intensity profiles [(K) and (L)]. Data are presented as means ± SEM. Nested *t* test [(B), (D), (F), (H), and (J)] was used. *P < 0.05, ****P < 0.0001; ns, not significant. au, arbitrary units. Details of replicates and number of neurons analyzed in tables S1 and S2. h, hours; d, days.

The most likely way for proteins like CK1δ to end up in the GVB core is via autophagy. To explore the role of autophagy in GVB formation, tau^+^ neurons were treated with Sar405 to block VPS34 (Fig. 4A and fig. S7A). This efficiently inhibited autophagic flux, as indicated by strongly increased levels of P62 (fig. S7B). In addition, the number of GVB^+^ neurons gradually decreased over time down to 22% of control after 8-day treatment (Fig. 4, A and B). To further support the involvement of autophagy in GVB formation, KD of *Fip200* and *Atg5*, which act after autophagy initiation, was performed (fig. S7A). *Fip200* as well as *Atg5* shRNAs (*51*, *52*) efficiently reduced the protein levels of their targets (fig. S7, C, D, F, and G) and both increased P62 levels (fig. S7, E and H), showing inhibition of autophagy. All shRNAs reduced the number of GVB^+^ neurons (*shFip200* to 39% and *shAtg5* to 25% of control) (Fig. 4, C to F). To test the effect of activation of autophagy, we used the mechanistic target of rapamycin (mTOR) inhibitors Torin1 and rapamycin for 48 hours (fig. S7A). Both treatments strongly reduced 4E-BP1 phosphorylation, showing efficient inhibition of mTOR activity (fig. S7, I and K). In contrast, the number of GVB^+^ neurons was not affected by either treatment (fig. S7, J and L). To study whether autophagic activity is increased in tau^+^/GVB^+^ neurons compared to tau^+^/GVB^−^ neurons, P62 levels were determined in the absence and presence of bafilomycin (BafA1) to determine autophagic flux. This shows that the autophagic flux is not different between tau^+^/GVB^−^ and tau^+^/GVB^+^ neurons (fig. S8, A and B). In addition, to measure proteasomal activity, we used the proteasomal activity probe Me4BodipyFL-Ahx3Leu3VS (*53*, *54*) in tau^+^ neurons. Treatment with the proteasome inhibitor MG132 decreased the intensity of the activity probe (fig. S8C), but no difference in probe intensity was observed between tau^+^/GVB^−^ and tau^+^/GVB^+^ neurons, indicating that there is no major difference in proteasome activity in tau^+^/GVB^−^ and tau^+^/GVB^+^ neurons (fig. S8, C and D). Together, these results demonstrate that GVB formation is dependent on the autophagic machinery and basal flux but is not stimulated by increasing autophagic activity.

These data support a role for autophagy in the sequestration of proteins, like CK1δ, in the GVB core after the initiation of the pathway. To investigate whether autophagy is also an upstream regulator of the pathway that makes neurons GVB^+^, we studied the connection between CK1δ and autophagy in GVB formation. To this end, we used the Dox-inducible expression system for GFP-CK1δ. Inhibition of autophagy with Sar405 after increasing CK1δ levels by the addition of Dox did not affect CK1δ accumulation in GVBs (Fig. 4, G and H). In line with this, stimulation of autophagy with Torin1 before treatment with CK1δ_i_ (Fig. 4I) did not rescue the CK1δ_i_-induced reduction in GVB^+^ neurons (Fig. 4J). The combined results place CK1δ activity upstream in the pathway leading to GVB^+^ neurons and suggest that autophagy sequesters CK1δ in the GVB after it has performed its role in the process.

Pathological tau does not accumulate in the GVB in the human brain and experimental models (*13*, *18*). To exclude that GVBs rapidly degrade tau, thereby precluding the detection of tau inside the GVB, lysosomal degradation was inhibited using E64D and pepstatin A. This strongly increased in the accumulation of P62 (fig. S9A), confirming effective lysosomal inhibition. However, this treatment did not result in the accumulation of AT8-positive tau in the GVB (Fig. 4K). In addition, whereas intraneuronal tau pathology was positive for the aggregate-binding dye Proteostat, no positive signal was observed in GVBs (Fig. 4L). This suggests that GVBs do not play a key role in pathological tau degradation.

### GVB^+^ neurons are resilient to tau-induced protein synthesis impairment

**Fig. 5.**
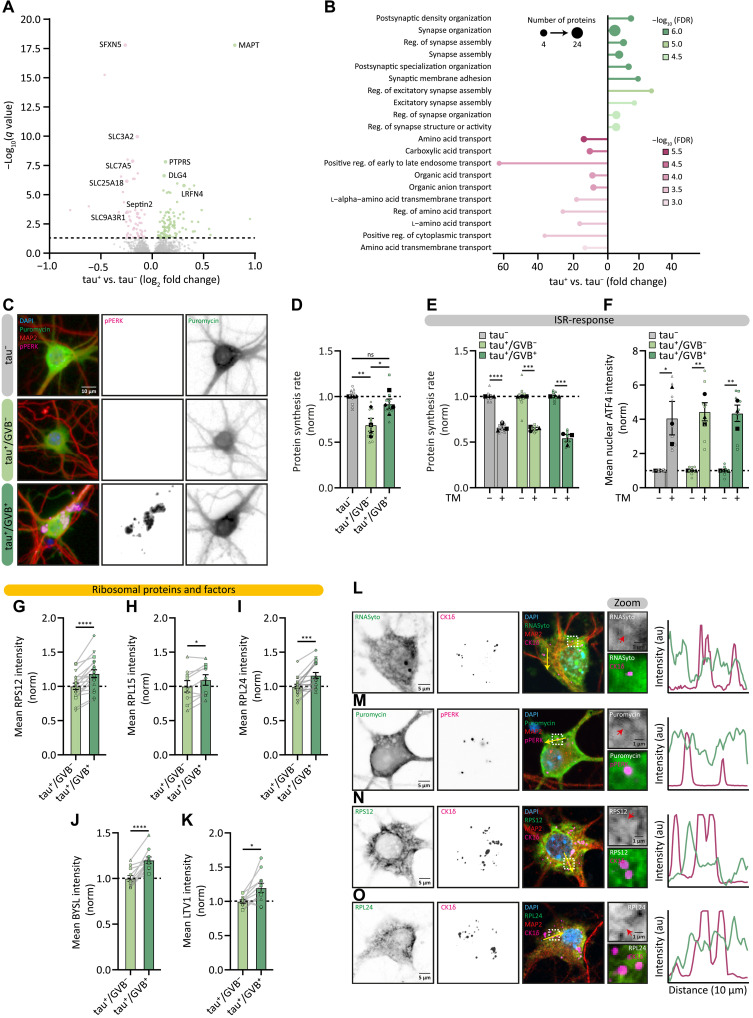
GVB^+^ neurons are resilient to tau-induced protein synthesis impairment. (**A** and **B**) Volcano plot (A) and GO enrichment analysis (B) showing significantly up-regulated (green) and down-regulated (pink) proteins (*q* value < 0.05) and GO terms, respectively, in tau^+^ neurons compared to tau^−^ neurons analyzed at day 15 (see table S3). (**C** to **E**) Analysis of tau^−^, tau^+^/GVB^−^, and tau^+^/GVB^+^ neurons treated for 15 min with puromycin before analysis at day 22. (C) Representative high-content microscopy images showing MAP2 (red), pPERK (magenta), and puromycin (green). (D) Puromycin intensity was quantified to assess protein synthesis rate normalized to tau^−^ neurons (*N* = 4; *n* = 12). (E) Protein synthesis rate w/wo TM for 24 hours, normalized to untreated for each group (*N* = 3; *n* = 8-9). (**F**) Mean nuclear ATF4 intensity w/wo TM for 24 hours, normalized to untreated for each group (*N* = 3; *n* = 9). (**G** to **K**) Mean somatic intensities in tau^+^/GVB^−^ and tau^+^/GVB^+^ (CK1δ based) neurons analyzed at day 22 for RPS12 (G), RPL15 (H), RPL24 (I), BYSL (J), and LTV1 (K) (*N* = 3 to 6; *n* = 9 to 18). (**L** to **O**) Representative confocal images of tau^+^/GVB^+^ neurons at day 22, showing SYTO RNASelect (green) and CK1δ (magenta) (L), puromycin (green) and pPERK (magenta) (M), RPS12 (green) and CK1δ (magenta) (N) and RPL24 (green) and CK1δ (magenta) (O) and MAP2 (red). Dashed squares indicate zoomed regions; yellow arrows indicate the location of the intensity profiles. Red arrows depict GVB cores. Nuclei are in blue, separate channels in grayscale [(C) and (L) to (O)]. Data are presented as means ± SEM. Nested one-way ANOVA followed by a Tukey’s post hoc test (D), nested *t* test [(E) and (F)] and paired *t* test [(G) to (K)] were used. *P < 0.05, **P < 0.01, ***P < 0.001, ****P < 0.0001; ns, not significant. au, arbitrary units. Details of replicates and number of neurons analyzed in tables S1 and S2.

To investigate alternative potential functional implications of the presence of GVBs, early proteome changes induced by tau pathology at day 15 were determined by comparing tau- and tau^+^ neurons (Fig. 5A and table S3). Gene ontology (GO) analysis indicated that the up-regulated functional processes primarily involved pathways affecting the synapse (Fig. 5B) in line with the tau/GVB model representing an early stage preceding neurodegeneration (fig. S1, A to L). The most significantly down-regulated functional protein groups are solute carrier membrane transport proteins, predominantly amino acid transporters (Fig. 5B). This may affect the availability of amino acids for the biosynthesis of proteins and is in line with reduced neuronal protein synthesis rates upon pathological tau accumulation (*2*–*6*). To directly determine de novo protein synthesis in relation to tau pathology and GVB state, we used the surface sensing of translation (SUnSET) puromycin labeling assay (*55*, *56*) combined with quantitative high-content automated microscopy to analyze tau^−^ and tau^+^ neurons at day 22. This assay assesses protein synthesis rates by measuring the incorporation of puromycin in newly synthesized proteins during a short pulse labeling. We developed a script to differentiate between tau^+^/GVB^−^ and tau^+^/GVB^+^ neurons within the tau^+^ population. Tau^+^/GVB^−^ neurons, which constitute most tau^+^ neurons, displayed a pronounced reduction in protein synthesis rates of 31% compared to tau^−^ neurons (Fig. 5, C and D). In contrast, tau^+^/GVB^+^ neurons retained protein synthesis rates similar to those of tau^−^ neurons (Fig. 5, C and D).

Next, we investigated whether these differences in protein synthesis were mediated by transient adaptations to proteostatic stress, of which the integrated stress response (ISR) is a key regulatory pathway. To test whether the GVB^+^ neurons actively maintained protein synthesis rates or were less responsive to ISR-induced protein synthesis reduction, neurons were treated with the ISR inducer tunicamycin (TM). TM treatment resulted in a strong reduction in protein synthesis and increased nuclear ATF4 intensity independent of the presence of tau pathology or GVBs (Fig. 5, E and F, and fig. S10A). This shows that tau^−^, GVB^−^, and GVB^+^ neurons can all dynamically adjust their protein synthesis levels in response to stress. Treatment with the ISR inhibitor ISRIB did not affect protein synthesis rates in GVB^−^ or GVB^+^ neurons (fig. S10B), further supporting that the ISR is not involved in GVB-associated protein synthesis resilience. Protein synthesis can also be transiently reduced by the mTOR pathway. However, there was no difference in the phosphorylation of the mTOR substrate 4E-BP1 neurons without and with tau pathology, suggesting that the mTOR pathway is not activated in the pool of tau^+^ neurons (fig. S10, C and D). Also, treatment with pharmacological inhibitors of the mTOR- and angiogenin-mediated stress pathways did not affect protein synthesis rates in tau^+^/GVB^−^ or tau^+^/GVB^+^ neurons (fig. S10, E and F). These data collectively show that transient stress pathways are not directly involved in the tau-induced protein synthesis reduction or the resilience against it in GVB^+^ neurons.

To determine whether adaptations in the protein synthesis machinery itself underlie the resilience, we analyzed the levels of ribosomal proteins and biogenesis factors in tau^+^ neurons. The levels of ribosomal proteins RPS12, RPL15, and RPL24 were increased in tau^+^/GVB^+^ neurons compared to tau^+^/GVB^−^ neurons by 18, 9, and 16%, respectively (Fig. 5, G to I). Also, the levels of ribosomal assembly factors BYSL and LTV were 20% higher in tau^+^/GVB^+^ neurons (Fig. 5, J and K). To investigate whether the GVB itself functions as a localized hub for protein synthesis, we assessed the colocalization of GVBs with key components of the translation machinery using confocal microscopy. We stained for RNA, using RNASyto Green, nascent proteins via puromycin pulse labeling and RPS12 and RPL24 (Fig. 5, L to O). No overlap was observed between these markers and GVBs, making it unlikely that GVBs themselves directly contribute to protein synthesis. These results suggest that GVB^+^ neurons elicit a response to ameliorate reduced protein synthesis upon tau aggregation that includes increased ribosomal content.

### GVB^+^ neurons retain the capacity to control LTP-regulated protein synthesis in the presence of tau pathology

**Fig. 6.**
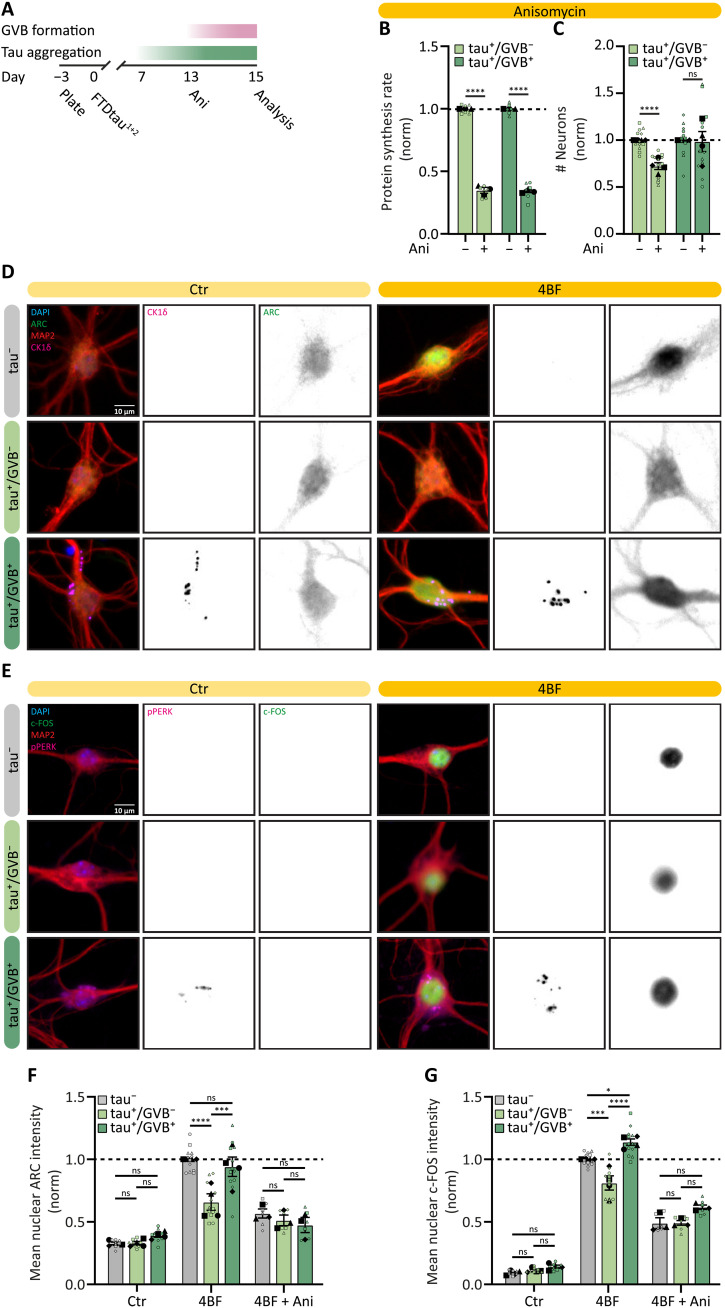
GVB^+^ neurons fully retain the capacity to control LTP-regulated protein synthesis in the presence of tau pathology. (**A**) Experimental timeline. (**B**) Puromycin intensity was quantified to assess protein synthesis rate upon 48 hour of Ani treatment in tau^+^/GVB^−^ and tau^+^/GVB^+^ (pPERK based) neurons and normalized to untreated per group (*N* = 3; *n* = 6). (**C**) Neuron number of tau^+^/GVB^−^ and tau^+^/GVB^+^ (pPERK based) neurons upon 48 hours of Ani treatment normalized to untreated per group (*N* = 4; *n* = 12). (**D** and **E**) Representative high-content microscopy images of tau^−^, tau^+^/GVB^−^, and tau^+^/GVB^+^ neurons treated w/wo 4BF for 4 hours, showing ARC (D) or c-FOS (E) (green), MAP2 (red), and CK1δ (magenta), and DAPI (blue). Separate channels are in grayscale. (**F** and **G**) Mean nuclear ARC (F) or c-FOS (G) in tau^−^, tau^+^/GVB^−^, and tau^+^/GVB^+^ [CK1δ (F) or pPERK (G) based] neurons w/wo 4BF treatment and w/wo Ani treatment before 4BF treatment, normalized to tau^−^ 4BF-induced response (*N* = 3 to 4; *n* = 8 to 12). Data are presented as means ± SEM. Nested *t* test [(B) and (C)] and nested one-way ANOVA followed by post hoc Sidak’s test [(F) and (G)] were used. *P < 0.05, ***P < 0.001, ****P < 0.0001; ns, not significant. Details of replicates and number of neurons analyzed in tables S1 and S2.

We showed that GVB^+^ neurons are resilient to tau-induced neurodegeneration (Fig. 1, E to G). To investigate whether the increased survival of GVB^+^ neurons is related to protein synthesis capacity, protein synthesis was inhibited using 48 hours of anisomycin treatment (Fig. 6A), which reduced protein synthesis to a similar extent in GVB- and GVB^+^ neurons, 65 and 66%, respectively (Fig. 6B). However, while anisomycin treatment resulted in synthetic lethality with tau pathology in GVB^−^ neurons (28% neuron loss), GVB^+^ neuron numbers were unaffected (Fig. 6C). These results suggest that GVB^+^ neurons are more resilient to toxicity induced by pharmacological protein synthesis inhibition.

To study the relevance of the resilience of GVB^+^ neurons for neuronal function, we assessed the capacity of tau^+^ neurons to regulate the acute synthesis of immediate early genes (IEGs) that are involved in the changes of synaptic strength, which is essential for learning and memory-related processes (*57*, *58*). To study whether tau aggregation also reduces LTP-related IEG induction in our model, treatment with 4BF was used. 4BF is a combination of 4AP (Kv1 channel blocker), bicuculline (γ-aminobutyric acid receptor antagonist), and forskolin (adenylyl cyclase activator) that has been shown to increase network activity and induce *N*-methyl-d-aspartate–dependent LTP in primary neuron cultures (*59*, *60*). 4BF-induced LTP rapidly increases the levels of the IEGs c-FOS and ARC (*59*). Neuronal plasticity was induced using 4BF for 4 hours after 15 days of tau exposure. Quantitative high-content automated microscopy was used to determine expression levels of ARC and c-FOS in the nucleus of tau^−^, tau^+^/GVB^−^, and tau^+^/GVB^+^ neurons. Both ARC and c-FOS expression were strongly increased upon the induction of chemical LTP (Fig. 6, D to G). In tau^+^/GVB^−^ neurons, ARC and c-FOS levels upon LTP induction were significantly lower than in tau^−^ neurons (decreased by 34 and 19%, respectively), while the tau^+^/GVB^+^ neurons do not differ or are even higher in ARC and c-FOS expression levels from tau^−^ neurons, respectively (Fig. 6, F and G). As expected, the up-regulation of ARC and c-FOS upon LTP induction was dependent on de novo protein synthesis as anisomycin reduced the increase of these proteins (Fig. 6, F and G, and fig. S11, A and B). These data show that GVB^+^ neurons are resilient to tau-induced impairment in acute LTP-regulated protein synthesis.

## DISCUSSION

**Fig. 7.**
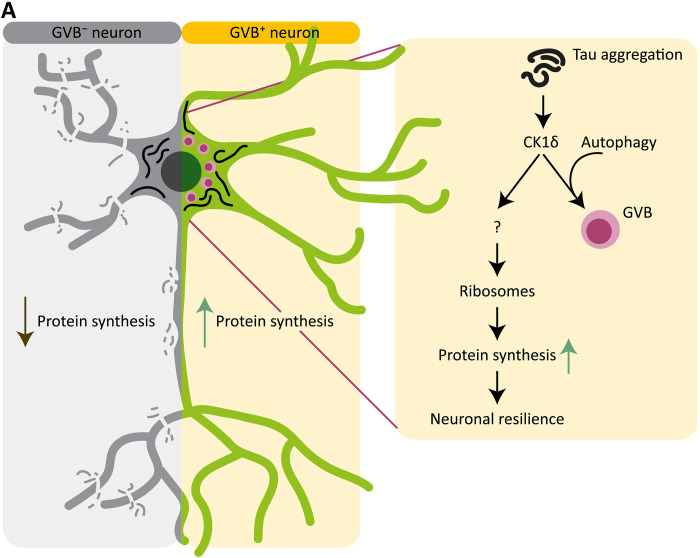
Model for the resilience to tau pathology–mediated neurodegeneration by GVB formation. (**A**) Intraneuronal tau aggregation impairs protein synthesis and ultimately leads to neuron loss. In a subset of neurons, CK1δ activation drives GVB formation in an autophagy-dependent manner. GVB^+^ neurons are resilient to tau-induced protein synthesis impairment and neurodegeneration. This resilience is facilitated by enhanced ribosomal content, which supports protein synthesis by increasing the protein synthesis machinery. This allows GVB^+^ neurons (in contrast to GVB^−^ neurons) to fully retain the capacity to induce the LTP-regulated synthesis of IEGs in the presence of tau pathology, unlike GVB^−^ neurons. Our data identify CK1δ-induced GVB formation as an intrinsic neuron-specific resilience mechanism that provides neuronal protection against tau pathology.

In response to pathological protein assemblies, including tau and α-synuclein, neurons can develop GVBs (*23*, *24*). In the human AD brain—like in our model—the surviving neurons during disease progression are increasingly GVB^+^ (*21*, *27*, *61*), in accordance with a protective rather than a degenerative role. Our findings demonstrate that CK1δ activity is essential for GVB formation. In addition, we show that autophagy is required for the sequestration of CK1δ into the GVB. The combined data support a model where GVB^+^ neurons have adapted to tau-induced reduction of protein synthesis rate, with GVBs as a marker of resilient tau-positive neurons (Fig. 7A).

Disturbed translational regulation and overall protein synthesis impairment are observed in models of tauopathies (*2*–*6*) and several other neurodegenerative proteinopathies (*62*–*67*). Transient reduction of protein synthesis upon disturbed proteostasis is a physiological protective mechanism that is an integral part of stress responses like the ISR and mTOR pathways (*68*–*70*). For example, during healthy aging in *Caenorhabditis elegans*, prolonged suppression of global protein synthesis by reducing ribosomal protein expression extends the lifespan (*71*). Similarly, stimulating ISR-mediated protein synthesis reduction before the onset of pathology rescues neurodegeneration in transgenic mouse models (*72*). In contrast, in the presence of existing pathology, neurodegenerative phenotypes are ameliorated by restoring protein synthesis (*5*, *67*, *73*–*75*), supporting the notion that impaired protein synthesis is considered an important pathogenic mechanism in many neurodegenerative diseases. Consistent with these observations, increased levels of ribosomes and translation factors have been associated with a resilience phenotype in amyotrophic lateral sclerosis, although this is not accompanied by detectable increases in protein synthesis rate in later stages of pathology (*76*). Prolonged reduction of protein synthesis in the presence of extensive protein pathology, as for example in human tauopathies, may affect neuronal viability and function by impairing the replacement of critical structural proteins but also by directly affecting synaptic plasticity, which requires adequately regulated de novo protein synthesis (*77*, *78*). The acute and dynamic demand for protein synthesis that is required for synaptic plasticity includes the rapid induction of IEG proteins. Tau pathology has been demonstrated to impair synaptic plasticity, as well as the expression of IEGs in animal models (*79*, *80*). Also in our model, tau aggregation reduces global protein synthesis rates as well as the LTP-regulated induction of IEGs c-FOS and ARC. c-FOS is synthesized in response to increased neuronal activity (*81*, *82*), and ARC is required for the consolidation of LTP and maintenance of synaptic strength (*83*, *84*), both essential for learning and memory-related processes (*57*, *58*). Our data demonstrate that GVB^+^ neurons not only retain global protein synthesis rates similar to those of neurons without tau pathology but also retain the capacity to adequately regulate LTP-related IEG protein expression. This demonstrates that GVB^+^ neurons are resilient to tau pathology-induced protein synthesis decline.

To identify the mechanism by which GVB^+^ neurons adapt to the tau-induced protein synthesis decrease, transient proteostatic responses mediated by the ISR, mTOR, and angiogenin were investigated. No evidence for their involvement in tau-induced reduction of protein synthesis or the difference between GVB^−^ and GVB^+^ neurons was found, suggesting that the rescue of protein synthesis involves a more long-term adaptation of the protein synthesis machinery. In AD, progressive ribosomal loss correlates with tau pathology (*85*), aligning with studies in tauopathy mouse models that demonstrate decreased ribosomal protein synthesis accompanied by reduced overall protein synthesis (*6*). Moreover, targeted laser capture proteomics of human AD hippocampus demonstrated that tangle-bearing neurons exhibit a loss of ribosomal proteins; however, in GVB^+^ neurons, the ribosomal protein levels are similar to those in neurons without tau pathology (*86*). We demonstrate here that GVB^+^ neurons have increased levels of the small ribosomal subunit protein RPS12, the large ribosomal subunit proteins RPL15 and RPL24 as well as the ribosomal biogenesis factors LTV1 and BLTV. In cell lines, CK1δ was previously demonstrated as a critical regulator of ribosome assembly through phosphorylation of LTV1 and BYSL (*35*–*37*). The role of CK1δ in ribosomal biogenesis in neurons is unknown and is a potential candidate for further investigation of GVB resilience in future studies. This shows that GVB^+^ neurons have multiple adaptations in the protein biosynthesis machinery that would allow them to counteract the tau pathology–induced decline in protein synthesis. Prolonged protein synthesis impairment by tau pathology will limit the ability to survive additional challenges to the protein synthesis machinery. In line with this, we observe synthetic lethality of pharmacological protein synthesis inhibition and tau pathology in GVB^−^ but not GVB^+^ neurons. The overall increased protein biosynthesis capacity in GVB^+^ neurons will result in a healthier proteome that is capable to combat the toxicity of short-term protein synthesis block in GVB^+^ neurons.

In addition to protein synthesis, an intricate network of chaperones and proteolytic systems underlies the maintenance of protein homeostasis (*87*). Our data do not show differences in the accumulation of pathological tau (*24*), proteasomal activity or autophagic flux between GVB^−^ and GVB^+^ neurons. Although GVBs are lysosomes, they do not notably contribute to the degradation of pathological tau aggregates since tau does not accumulate within GVBs. GVBs have degradative capacity similar to the non-GVB lysosomes in the same cell but are uniquely characterized by the presence of one or multiple dense cores (*23*). Live-cell imaging and half-life calculations show that these GVB cores are highly stable. The accumulation of proteins in the GVB core depends on the autophagic machinery, but inhibition of autophagy does not result in accumulation of GVB cores in the cytosol, suggesting that the cores either form within autolysosomes or originate in the cytosol but are rapidly degraded if not engulfed. The basal autophagic machinery is sufficient for the formation of GVBs. CK1δ has been implicated in the early steps of autophagy (*40*–*42*, *88*), potentially explaining why CK1δ overexpression can rescue the partial reduction of GVB formation by 48 hours of Sar405 treatment. Although CK1δ activity is necessary for the formation of GVB^+^ neurons, catalytic activity in cis is not required for localization to GVBs. The kinase-dead mutant CK1δ-K38M, expressed in the presence of endogenous CK1δ, does not function as a dominant negative. In the presence of CK1δ-K38M, endogenous CK1δ activity is sufficient to drive GVB formation, while the inactive mutant still localizes to the GVB. This highlights the dual aspects of the involvement of CK1δ in GVBs: activity-dependent induction of GVB formation versus activity-independent accumulation in the GVB core. These combined data support a model where the accumulation of CK1δ in GVBs occurs by autophagy during the formation of GVB^+^ neurons. Previous studies showed that CK1δ activity is tightly controlled to prevent dysregulation of the enzyme (*33*, *89*). The strongly increased levels of CK1δ upon inhibition of its activity we observe suggests that neurons also require tight regulation of CK1δ. Therefore, it is not unexpected that there is a direct coupling between the function of CK1δ in initiating the neuronal resilience to tau pathology in GVB^+^ neurons and the prevention of persisting or excessive activation by sequestering CK1δ in GVBs. The prominent presence of CK1δ in the core could indicate a negative feedback mechanism to sequester the key regulator of the protective mechanism in GVB^+^ neurons to prevent overactivation of the pathway. Currently, the lack of interventions that can disconnect the resilience program in GVB^+^ neurons and the formation of GVBs prevents straightforward investigation of the mechanistic relation between CK1δ and the protein synthesis phenotypes. This also complicates a more definite conclusion that GVBs are merely a by-product of activation of the protective mechanism in GVB^+^ neurons. However, currently, there is no indication for a specific function associated with GVBs themselves.

Follow-up studies should address how tau aggregates induce CK1δ activity and what substrates are involved in resilience. The number of neurons with tau pathology that become GVB^+^ increases over time, suggesting a (partially) stochastic process. CK1δ is traditionally considered a constitutively active protein, relying on binding partners or scaffold proteins for subcellular localization (*33*, *90*), although also differential autophosphorylation was more recently shown to regulate substrate selectivity (*91*). CK1δ binds to and phosphorylates tau at different sites (*33*). Tau assemblies may provide a platform for CK1δ binding and signaling, leading to GVB formation. This may involve structural rather than primary sequence motifs because GVBs also form in response to α-synuclein aggregates (*24*), although the involvement of CK1δ and protein synthesis resilience in α-synuclein–induced GVBs has currently not been demonstrated.

The identification of specific molecular targets to stimulate GVB formation has therapeutic potential. Because GVBs form selectively in neurons, it is likely that, in addition to CK1δ, cell type–specific factors and additional signaling pathways contribute to their resilience. In our model the transduction efficiency of CK1δ was insufficient to directly assess its effectivity to rescue neurodegeneration. Regardless, the involvement of CK1δ in a great diversity of cellular processes limits its utility as therapeutic target. Ultimately, combining resilience-boosting therapy with tau-reducing therapies like *MAPT* antisense oligonucleotides or tau immunotherapy is an attractive scenario to halt the disease while also protecting neurons against the cellular effects of tau pathology to help them to retain function. By elucidating the mechanistic underpinnings of the formation and functional implications of the presence of GVBs in neurons, our work offers insight into neuronal resilience in tauopathies.

## MATERIALS AND METHODS

### Animals and primary cell culture

All animal experiments are approved by the “Centrale Commissie Dierproeven” (Central Commission for Animal Experiments) of the Netherlands government and will be performed according to the Netherlands Law on Animal Research (Wet op de dierproeven) in full agreement with the Directive 2010/63/EU with local approval by and under supervision of the Animal Welfare Body Amsterdam UMC - VU. C57BL/6 mice were bred in-house or obtained from Charles River.

Embryonic day 18.5 (E18.5) wild-type (WT) mouse embryos or postnatal day 1 (P1) mice were dissected to obtain cerebral cortices after disposal of the meninges. The cortices were digested in Hanks’ balanced salt solution (Sigma-Aldrich) containing 10 mM Hepes (Gibco; Hanks-Hepes) and 0.25% trypsin (Gibco) for 20 min at 37°C. Hereafter, the digested tissue was washed twice with Hanks-HEPES and subsequently with Dulbecco’s modified Eagle’s medium (DMEM, Lonza) containing 10% heat-inactivated fetal bovine serum (Gibco), 1% penicillin-streptomycin (Gibco), and 1% nonessential amino acid solution (Gibco; DMEM^+^) in which the tissue was triturated using a fire-polished Pasteur pipette. Subsequently, the dissociated cells were spun down for 5 min at 800*g* at room temperature (RT), resuspended and plated in neurobasal medium (Gibco) supplemented with 2% B-27 (Gibco), 18 mM Hepes, 0.25% Glutamax (Gibco), and 0.1% penicillin-streptomycin (NB^+^).

Primary cortical neurons were cultured in plates and glass coverslips coated with poly-l-ornithine (5 μg/ml; Sigma-Aldrich) and laminin (2.5 μg/ml; Sigma-Aldrich and Bio-Techne) overnight (O/N) at RT. All cells were maintained at 37°C and 5% CO_2_. For Western blotting, cortical neurons were plated in a six-well plate at a density of 300,000 neurons per well. For immunolabeling, neurons were plated on 13-mm glass coverslips or in a 96-well plate (Greiner) at a density of 40,000 cells or 12,500 cells per well, respectively. After 10 days in vitro (DIV10), fresh NB^+^ was supplied to each well at 40% of the total volume in the well.

### Lentiviral transduction in primary cells

Lentiviral transduction with the human 2N4R P301L, S320F tau (FTDtau^1+2^), and FTDtau^1+2^ with an in-frame C-terminal enhanced GFP (EGFP) tag (FTDtau^1+2^-GFP) were performed on DIV3 (day 0), unless otherwise specified, in primary neurons (*24*–*26*). WT mouse CK1δ (a gift from Y.E. Greer) has been described before (*92*), and the formation of N-terminally tagged CK1δ with EGFP (GFP-CK1δ) has been described before (*23*). GFP-CK1δ_RES_ was changed from WT CK1δ in the *shCsnk1d* target sequence to prevent KD (Table 1) (new sequence: CGGGA**C**CG**T**GA**G**GAACGATTA). An NES was N-terminally cloned into the GFP-CK1δ construct. For the kinase-dead variant of CK1δ, a single point mutation in Lys^38^ to Met^38^ (AAG to ATG) was created to form GFP-CK1δ-K38M. As a control for EGFP-tagged constructs, a lentiviral cytomegalovirus (CMV)–driven EGFP construct was used. All constructs were subcloned into lentiviral backbone vectors under the CMV, the neuron-specific synapsin (Syn), or a Dox-inducible Syn promoter using the Gateway system (Invitrogen). The production of lentiviral particles was performed as described previously (*93*), and the lentiviral particles were stored at −80°C until use.

**Table 1.** The names, targeting gene, and sequence of the shRNA of all shRNAs used in this research. All lentiviral constructs were transduced on DIV10 (day 7), except FTDtau^1+2^ (-GFP) (specified in the text), *shCsnk1d-1* and the *shScr* as control on DIV14 (day 11) and GFP-CK1δ_RES_ and GFP as control on DIV7 (day 4).

Name	Gene	Sequence
*shScr*	-	CCGCAGGTATGCACGCGT or TTCTCCGAACGTGTCACGT
*shCsnk1d*	CK1δ	CGGGATCGAGAAGAACGATTA
*shFip200*	FIP200	GAGAGAACTTGTTGAGAAA
*shAtg5*	ATG5	GCAGAACCATACTATTTGCTT

Plasmids for shRNA constructs for the scrambled control (*shScr*) and CK1δ (Sigma-Aldrich; TRCN0000023773) were obtained from the MISSION shRNA library (Sigma-Aldrich). shRNAs targeting FIP200 (a gift of M. Kuijpers) (*51*) and ATG5 were cloned using oligonucleotides (Sigma-Aldrich) based on previously published research (*52*). The shRNAs were cloned into a second-generation lentiviral backbone under a CMV promoter.

### Cell treatments

Primary neurons were treated with 50 μM E64D and 20 μM pepstatin-A (Sigma-Aldrich) for 24 hours. Sar405 (5 μM; Sigma-Aldrich) was used for indicated times to inhibit VPS34 and 5 μM Torin1 (Bioke) (72, 48, or 24 hours) or 1 μM rapamycin (Hello Bio) (48 hours) were used to inhibit mTOR. Bafilomycin A1 (100 nM, Sigma-Aldrich) was used for 4 hours to block autophagic degradation. Proteasome activity was measured using proteasome probe Me4BodipyFL-Ahx3Leu3VS, which has been described previously (*53*, *54*). The probe was added to the media at 500 nM for 1 hour before fixation. Neurons were preincubated with MG132 at 10 μM 1 hour before adding the probe. To inhibit CK1δ, neurons were treated with PF-670462 (CK1δ_i_, TargetMol) for indicated times at a concentration of 12.5 μM. To induce ER stress, primary neurons were treated with TM (Sigma-Aldrich) for 24 hours at 5 μg/ml. Two hundred nanomolar of the ISR inhibitor ISRIB (Selleck Chemicals) was added 2 hours before fixation. Angiogenin inhibitor (NSC-65828, National Cancer Institute) was added 24 hours before fixation at a final concentration of 75 mM and was previously validated (*56*). Anisomycin (Sigma-Aldrich) was added for 48 hours, or directly before the induction of LTP, at a final concentration of 50 ng/ml to determine the effect on global protein synthesis. To label de novo synthesized proteins, we performed SUnSET as previously described (*55*). In short, primary cells were incubated with 2 μM puromycin (InvivoGen) for 15 min before fixation. Using the antipuromycin antibody (see Table 2), puromycinylated proteins were detected. To stain nucleic acids, the cells were incubated with 1 μM SYTO RNASelect (Thermo Fisher Scientific) for 4 hours before methanol fixation. Compounds were dissolved in H_2_O (puromycin) or dimethyl sulfoxide (the rest) and diluted in NB^+^. Equal amounts of solvent were added to the control conditions or when multiple concentrations were used, the highest concentration was used to determine the amount of vehicle added.

**Table 2.** Antibodies used in this research. From left to right: The target protein the primary antibody was raised at; for antibodies targeting specific phosphor-sites, the phospho-epitope is listed; the species of the primary antibody; the dilution used for immunofluorescence (IF) or Western blot (WB); the source; the product number.

Antibody	Phospho-epitope	Species	Dilution		Source	Product number
			IF	WB		
AT8	Ser^202^/Thr^205^	Mouse monoclonal	1:400		Thermo Fisher Scientific	MN1020
ATG5		Rabbit polyclonal		1:1000	Proteintech	10181-2-AP
ARC		Rabbit polyclonal	1:500		Synaptic Systems	156 003
BYSL		Rabbit polyclonal	1:250		Thermo Fisher Scientific	PA5-56679
c-FOS		Rat monoclonal	1:1000		Synaptic Systems	226 017
CK1δ		Mouse monoclonal	1:1000	1:2000	Santa Cruz Biotechnology	sc-55553
FIP200		Rabbit monoclonal		1:1000	Cell Signaling Technology	12436S
GOLGA4		Rabbit polyclonal	1:2000		Sigma-Aldrich	HPA0351021
GFP		Rabbit polyclonal		1:500	Bio-Connect	GTX20290
LAMP1		Rat monoclonal	1:500		BioLegend	121601
LAMP1		Rat monoclonal	1:1000		Abcam	ab25245
LTV1		Rabbit polyclonal	1:250		Thermo Fisher Scientific	PA5-56373
MAP2		Chicken polyclonal	1:500		Abcam	ab5392
MC1		Mouse monoclonal	1:500		Kind gift from P. Davies	
pPERK	Thr^981^	Rabbit polyclonal	1:1000		Santa Cruz Biotechnology	sc-32577
pPERK	Thr^982^	Rabbit polyclonal	1:500 - 1:1000		Thermo Fisher Scientific	PA5-102853
Puromycin		Mouse monoclonal	1:500		Milipore	MABE343
Puromcyin		Mouse monoclonal	1:3000		Kerafast	EQ0001
P62		Rabbit polyclonal	1:500	1:2000	Thermo Fisher Scientific	PA5-20839
RPL15		Rabbit polyclonal	1:250		Proteintech	16740-1-AP
RPL24		Rabbit polyclonal	1:250		Proteintech	17082-1-AP
RPS12		Rabbit polyclonal	1:250		Proteintech	16490-1-AP
SYP1		Guinea pig polyclonal	1:1000		Synaptic Systems	101004
4E-BP1		Rabbit monoclonal		1:1000	Cell Signaling Technology	9644
p-4E-BP1	Thr^37^/Thr^46^	Rabbit monoclonal		1:1000	Cell Signaling Technology	2855

### Induction of chemical LTP

Neurons at DIV18 (day 15) were stimulated for 4 hours, after which they were directly fixed. A cocktail of chemicals was used to induce network activity and plasticity for generation of IEG expression. The 4BF cocktail consists of 100 μM 4-aminopyridine (Sigma-Aldrich, A78403), 50 μM bicuculline (Sigma-Aldrich, 14343), and 50 μM forskolin (Sigma-Aldrich, 344282) as previously described (*59*).

### Fixation and immunolabeling of primary neurons

On DIV18 (day 15), DIV25 (day 22), or DIV32 (day 29) neurons were fixed in 1.85% formaldehyde (FA, Electron Microscopy Sciences) in phosphate-buffered saline (PBS) (pH 7.4) by the addition of equal volumes of 3.7% FA to the culture medium for 10 min followed by fixation in 3.7% FA for 10 min at RT. Primary neurons shown in Fig. 5M and fig. S4V were fixed in ice-cold methanol for 15 min on ice to remove soluble proteins. After fixation, the cells were washed with PBS and stored at 4°C in PBS (pH 7.4) in the dark until further use. Fixed cells were permeabilized in 0.5% Triton X-100 (Thermo Fisher Scientific) in PBS (pH 7.4) for 5 min at RT and blocked in 2% normal goat serum (Gibco) and 0.1% Triton X-100 in PBS (blocking solution) for 30 min at RT. Primary antibodies were diluted in blocking solution and incubated O/N at 4°C in the dark. The primary antibodies used and their dilutions are listed in Table 2. After three PBS washes, the cells were incubated with Alexa Fluor (405, 488, 546, 568, and 647)–conjugated secondary antibodies (Abcam and Thermo Fisher Scientific) in a 1:500 dilution in blocking solution for 1 hour at RT in the dark. The cells were subsequently washed thrice with PBS. Aggresomes were visualized using the proteostat aggresome detection kit (Enzo) according to the manufacturer’s details and washed three times with PBS. Hereafter, to visualize nuclei, the cells were incubated with 4′,6-diamidino-2-phenylindole (DAPI, Brunschwig Chemie) diluted in PBS (5 μg/ml) at RT for 5 min, followed by two PBS washes. Coverslips were mounted on microscope slides (Thermo Fisher Scientific) using Mowiol (Sigma-Aldrich) and were kept in the dark O/N at RT to dry. The slides were stored in the dark at 4°C until imaging. Ninety-six–well plates were stored in the dark at 4°C until imaging with the wells filled with 100 μl of PBS.

### Confocal microscopy and confocal image analysis

The mounted coverslips were imaged using a Nikon Eclipse Ti confocal microscope controlled by NisElements 4.30 software (Nikon), equipped with a 60× oil immersion objective [numerical aperture (NA) = 1.4] or a 40× oil immersion objective (NA = 1.3). Z-stacks with a step size of 0.25, 0.5, or 1 μm were obtained. Laser settings were adjusted to reduce saturation and kept the same in every independent experiment between conditions.

### Live microscopy

The cells were placed in a Tokai Hit live-cell microscope incubation system mounted in the Nikon Eclipse Ti confocal microscope, equipped with a 40× oil immersion objective (NA = 1.3) or a 10× air objective (NA = 0.45) for calcium imaging. Ten minutes before calcium imaging, 0.5 μM X-Rhod-1 (Thermo Fisher Scientific) was added to the cells. A video with 8 fps was made for the duration of 5 min. For following GVB dynamics, every 10, 20, or 30 min for a period of minimally 9 hours, a Z-stack with a step size of 1 μm was obtained. When FRAP was applied, a specified region in the GVB^+^ soma was photobleached and followed over the same period as described in the Results section.

Confocal images were analyzed using ImageJ software (National Institutes of Health). Neurons with two or more CK1δ or pPERK bright punctae were defined as GVB^+^ neurons and neurons without any punctae were defined as GVB^−^ neurons. Single confocal planes are shown unless otherwise stated.

Line segments were drawn to determine the overlap between multiple markers. The line graphs show fluorescence intensity in arbitrary units (AU).

### High-throughput microscopy and automated analysis

Cells cultured in a 96-well plate were imaged using CellInsight CX7 High-Content Screening (HCS) Platform (Thermo Fisher Scientific) controlled by HCS Studio Cell Analysis software (Thermo Fisher Scientific). Air objectives (10×, 20×, and 40×) were used in wide-field mode. The 10× or 20× objectives were used to determine toxicity of the treatment compared to control. The 20× objective was used for determination of morphology. The 20× and 40× objectives were used to measure intensity and determine the number of GVB^+^ neurons. A single focal plane was obtained for all channels. Twenty-five or 30 fields, distributed throughout the well, were imaged for 10× or 20× and 40×, respectively. In all cases, DAPI signal was used for autofocus for every image individually. On average, 10× imaging obtained data from 1000 to 10,000 neurons per well, 20× imaging from 50 to 1000 neurons per well, and 40× 25 to 500 neurons per well.

The data obtained from the high-throughput microscopy were analyzed with Columbus analysis software (v2.5.2.124862; PerkinElmer) by in-house–developed scripts. Each script was optimized per independent experiment. In every analysis, neuronal nuclei were defined by overlap between the nuclear marker DAPI and the neuron-specific marker MAP2 as well as the size and roundness of the nucleus. Outcome parameters were averaged per well (when applicable) and normalized to the mean of the control wells. These values were used in the analysis.

### Neuronal morphology

Dendritic morphology and presynaptic density were determined using in-house–developed scripts, previously published (*26*, *56*). In short, on the basis of MAP2 staining, dendritic morphology was determined using a built-in CSIRO neurite analysis module. Morphological features, including dendrite length, number of extremities, segments, and primary dendrites were normalized to the number of neuronal nuclei within that well. The amount of synaptophysin 1 (SYP1)–positive presynapses was determined on the basis of local intensity maxima within an enlarged MAP2 mask. The presynapse density was determined by dividing the total amount or SYP1 puncta over total dendrite length.

### Tau intensity in MAP2 mask

The intensity measurements of multiple readouts of tau aggregation, GFP-tagged, and untagged FTDTau^1+2^, were performed by measuring the sum of the intensity of FTDTau^1+2^-GFP or the conformational pathogenic tau marker MC1 in a MAP2 mask. The absolute intensity was normalized to the total area of the MAP2 mask.

### GVB selection

GVB^+^ neurons were defined using an altered protocol, as previously described (*23*, *24*). In short, the GVB marker (pPERK, CK1δ, Dox-GFP-CK1δ, or GolginA4) was subjected to a filter step to decrease background intensity using a curvature optimized per marker. Single puncta were selected on the basis of intensity. The single puncta were subsequently clustered on the basis of their proximity to one another. These clusters were defined as GVB clusters based on their size, roundness, intensity of the GVB-marker, the intensity of MAP2 in the cluster, and exclusion of the cluster within the nucleus based on DAPI signal. The percentage of GVB clusters was calculated by dividing the number of GVB clusters by the number of neurons in the same well.

### Somatic and nuclear intensity

The soma was defined as the MAP2-positive signal 13 μm around the neuronal nucleus (excluding overlap between bordering somas), after smoothing and filling the holes of the MAP2 signal. Subsequently, somas in neurons transduced with FTDtau^1+2^ were defined as GVB^−^ or GVB^+^ as previously described. False positives were excluded in tau^−^ neurons. Within each soma, the mean intensity of the marker (e.g., puromycin, RPS12, RPL15, RPL24, LTV1, and BYSL) was measured in the complete soma (cytosol and nucleus). Somatic ubiquitin intensity was background. Somatic CK1δ levels were determined by measuring CK1δ intensity in the soma without the GVBs. Nuclear intensity for CK1δ was corrected by subtracting the mean nuclear intensity by the mean intensity level of the cytosol without the nucleus. When determining CK1δ levels in a GVB^+^ neuron, pPERK was used to define GVB positivity. The nuclear intensity of c-FOS and ARC were not background corrected. The number of GVB^+^ somas was divided by the number of somas to calculate the percentage of GVB^+^ somas.

### GVB selection in GFP-positive neurons

For the selection of GFP-positive (GFP^+^) neurons, the soma was defined as previously described. Mean GFP intensity was measured in the complete soma region, including the nucleus. On the basis of an untransduced negative control, a threshold was set to select neurons with a higher somatic mean GFP intensity than the negative control. These neurons were defined as GFP^+^ neurons. GVB clusters were defined as GFP^+^ if they were localized within the GFP^+^ soma, creating GVB^−^/GFP^+^ and GVB^+^/GFP^+^ somas. Within these two populations, mean GFP intensity was measured in the complete soma (cytosol and nucleus), only the nucleus and the cytosol without the nucleus. Nuclear intensity was corrected by subtracting the mean nuclear intensity by the mean intensity level of the cytosol excluding the nucleus and GVBs, per neuron. The number of GVB^+^/GFP^+^ neurons was divided by the number of GFP^+^ neurons to calculate the percentage of GVB^+^/GFP^+^ neurons.

### Primary neuron lysis and Western blot analysis

The cells were washed two times with ice-cold PBS, and samples for the isolation of total proteins were scraped in loading buffer containing dithiothreitol (DTT, 50 mM) and 2% SDS to lyse the cells. The samples were triturated by a 21-gauge needle to sheer the DNA. For analysis of cytosolic proteins, the cells were lysed in 1% Triton X-100 (Thermo Fisher Scientific) in PBS supplemented with cOmplete protease inhibitor cocktail (Roche) and PhosSTOP phosphatase inhibitors (Roche). To purify these whole-cell lysates, the lysates were centrifuged at 14,000*g* for 10 min at 4°C. The total protein content of the supernatant was determined with the Pierce BCA protein assay kit (BCA) protein assay (Thermo Fisher Scientific). All samples were boiled for 5 min at 95°C. An equal volume or amount of the samples was loaded to a 4 to 15% Mini-Protean TGX stain-free precast polyacrylamide gels (Bio-Rad) and Precision Plus ProteinTM All Blue Standards (Bio-Rad) was used as a ladder. The total amount of protein on the gel was visualized using the Gel Doc EZ System (Bio-Rad) and analyzed using Image LabTM 6.0 (Bio-Rad). The total protein content was determined by correcting for the measured area size. The samples were then transferred onto 0.2 μM nitrocellulose membranes with the TransBlot Turbo transfer system and kit (both from Bio-Rad). The membranes were then blocked with 5% skimmed milk powder (Millipore) in tris-buffered saline containing 0.05% Tween (TBS-T) (Sigma-Aldrich) for 30 min at RT. Afterward, the membranes were incubated with primary antibody diluted in blocking buffer overnight at 4°C (See Table 2 for the dilutions of the primary antibodies). Subsequently, the membranes were washed four time for 15 min in TBS-T, whereafter the membranes were incubated with horseradish peroxidase–conjugated secondary antibodies (DAKO) diluted in blocking buffer (1:2000) for 1 hour at RT. Last, the membranes were washed four time for 15 min with TBS-T and submerged with SuperSignalTM West Femto Maximum Sensitivity Substrate kit (Thermo Fisher Scientific). Membranes were stripped for reblotting using ReBlot Plus Strong Antibody Stripping Solution (Merck). The chemiluminescence was visualized with the Odessey Fc system (Li-Cor) and analyzed using Image Studio 6.0 software (Li-Cor). Band intensities were corrected for the background signal.

### Proteomics analysis of primary neurons

#### Sample preparation

Primary neurons were untransduced (tau^−^) or transduced with FTDtau^1+2^ lentivirus (tau^+^) to induce tau aggregation. At day 15, the media were aspirated and neurons were subjected to 2× gentle ice-cold PBS washes. Neurons were then collected into 1.5-ml tubes using ice-cold PBS supplemented with protease inhibitor (Roche). The samples were centrifuged for 5 min at 3000*g* at 4°C, and the remaining pellet was resuspended in 7 μl of 5× loading buffer containing 10% SDS, 0.25 M tris (pH 6.8), 0.1% bromophenol blue, 0.5 M DTT, and 50% glycerol. The samples were stored at −20°C until further use. A total of 14 samples from two independent experiments were used per condition.

#### Protein in-gel digestion

Cell lysates were incubated at 98°C for 6 min to denature the proteins followed by incubation with 30% acrylamide/bis solution 37.5:1 (Bio-Rad) for 30 min at RT to block cysteine residues. The samples were loaded onto 1-mm-thick acrylamide gels. They were composed of 10% acrylamide, 0.375 M tris-HCl (pH 8.8), ultrapure H_2_O, 0.1% (w/v) ammoniumpersulfate and 6 μl of *N*,*N*,*N*′,*N*′-tetramethylethylene-diamine (Bio-Rad) per gel. Proteins were allowed to migrate into the gel by electrophoresis (120 V) for approximately 10 min. Peptides were extracted as previously described (*94*). Briefly, the gels were fixed O/N in a solution containing 50% (v/v) ethanol and 3% (v/v) phosphoric acid in H_2_O at RT and stained with colloidal Coomassie Blue (34% (v/v) methanol, 3% (v/v) phosphoric acid, 15% (w/v) ammonium sulfate, and 0.1% (w/v) Coomassie brilliant blue G-250 (Sigma-Aldrich). Sample-containing lanes were separated and cut in blocks. Gel fragments were destained in 50 mM NH_4_HCO_3_ (99% NH_4_HCO_3_, Fluka) and 50% (v/v) acetonitrile (high-performance liquid chromatography grade; JT Baker), dehydrated using 100% acetonitrile, reswell in 50 mM NH_4_HCO_3_ containing trypsin (10 μg/ml, sequence grade; Promega) and incubated O/N in a humidified chamber at 37°C to facilitate protein digestion. Peptides were extracted with a solution containing 0.1% (v/v) trifluoroacetic acid (Sigma-Aldrich) and 50% (v/v) acetonitrile. Samples were dried using a SpeedVac (Eppendorf) and stored at −20°C until liquid chromatography–mass spectrometry (LC-MS) analysis.

#### LC-MS analysis

Samples (75 ng) were loaded into EvoTips (EV2003; Evosep) and run on a 15 cm by 75 μm, 1.9 μm Performance Column (EV112; Evosep) using the Evosep One LC system with 30 samples per day program. Peptides were electro-sprayed into the TimsTOF Pro 2 mass spectrometer (Bruker Daltonics) and analyzed with diaPASEF (*95*). The MS was operated with the following settings: scan range 100 to 1700 mass/charge ratio (*m*/*z*), ion mobility 0.6 to 1.6 Vs/cm^2^, ramp time 100 ms, accumulation time 100 ms, and ramp rate 9.42 Hz.

#### Data analysis

Raw diaPASEF data from the TimsTOF Pro 2 were searched with a virtual spectral library generated from the UniProt mouse proteome UP000000589_1090 using DIA-NN 1.8.1 (*96*). Deep learning–based spectra and cross-run normalization were activated. The maximum number of missed cleavages was set to 1. The peptide length range was 7 to 30, the precursor charge range to 2 to 4, the precursor *m*/*z* range to 300 to 1800, and the fragment ion *m*/*z* range to 200 to 800. A fixed modification of UniMod: 24, 71.0371 at C was used, which represents acrylamide adduct. The precursor false discovery rate (FDR) was 1% (default). The rest of the settings were used as default.

MS-DAP (v. 1.0) (*97*) was used for quality control and candidate discovery on the results obtained from DIA-NN. Only peptides identified and quantified in at least six samples from each group were included in the analysis. The batch effect of samples from different experiments was removed by including the variable “batch” in MS-DAP. Normalization of peptide abundance values was applied, and the MSqRob algorithm was used for differential analysis at the peptide level using an FDR threshold of 5%.

GO and pathway analysis was performed using ShinyGO (v. 0.81) (*98*) with an FDR-adjusted *P* value threshold of 0.05. All detected proteins were used as background dataset. The glial genes GFAP, OLIG1, OMG and AQP4 (as shown by squares in Fig. 5A) were omitted for the GO-term analysis. A minimum of three proteins belonging to the same pathway and a maximum of 500 were considered to identify that pathway as a hit. The MS proteomics data have been deposited to the ProteomeXchange Consortium via the PRIDE (*99*) partner repository with the dataset identifier PXD061887.

### Statistical analysis

For each experiment, at least three biological replicates were performed, containing multiple technical replicates (specific number for experiments indicated by *N* and *n*, respectively, in the figure legends and table S1). The raw data are presented in table S2. Biological replicates are indicated in large, black shapes in the figures, while technical replicates belonging to the same biological replicate are smaller, lighter, and of the same shape, except in Fig. 1, E to G, for visual clarity. Data were normalized to the mean of the control condition, per time point unless otherwise specified. GraphPad Prism 8.4.2 software was used for statistical analysis. Visual, technical outliers in the pooled data were tested using the ROUT method (*Q* = 1%) and excluded when significant. A nested *t* test was used for the comparison of two groups or a nested one-way analysis of variance (ANOVA) followed by either a Dunnett’s or a Sidak’s multiple comparison post hoc test for the comparison of more than two groups (*100*). When only tau^+^/GVB^−^ and tau^+^/GVB^+^ neurons from the same well were compared, a paired *t* test was used. For Western blot analyses, an unpaired *t* test was used to compare two groups or a one-way ANOVA followed by a Sidak’s post hoc test was used when applicable. Only the most relevant *P* values were included in the figures. A *P* value of < 0.05 was considered statistically significant. *P < 0.05, **P < 0.01, ***P < 0.001, ****P < 0.0001, and ns.
