# Supplementary material for: Neurons with granulovacuolar degeneration bodies are resilient to tau-induced protein synthesis impairment
Source: Sci Adv. 2026 Mar 6;12(10):eaea8940. doi: 10.1126/sciadv.aea8940 (PMC12965320; doi:10.1126/sciadv.aea8940)
Supplement: Supplementary file 1 — Figs. S1 to S11 Legends for tables S1 to S3 Legend for movie S1 [file sciadv.aea8940_sm.pdf]

Science Advances
AAAS

Supplementary Materials for

Neurons with granulovacuolar degeneration bodies are resilient to tau-induced protein synthesis impairment

Jasper F. M. Smits *et al.*

Corresponding author: Wiep Scheper, w.scheper@amsterdamumc.nl

Sci. Adv. **12**, eaea8940 (2026)
DOI: 10.1126/sciadv.aea8940

The PDF file includes:

Figs. S1 to S11
Legends for tables S1 to S3
Legend for movie S1

Other Supplementary Material for this manuscript includes the following:

Tables S1 to S3
Movie S1

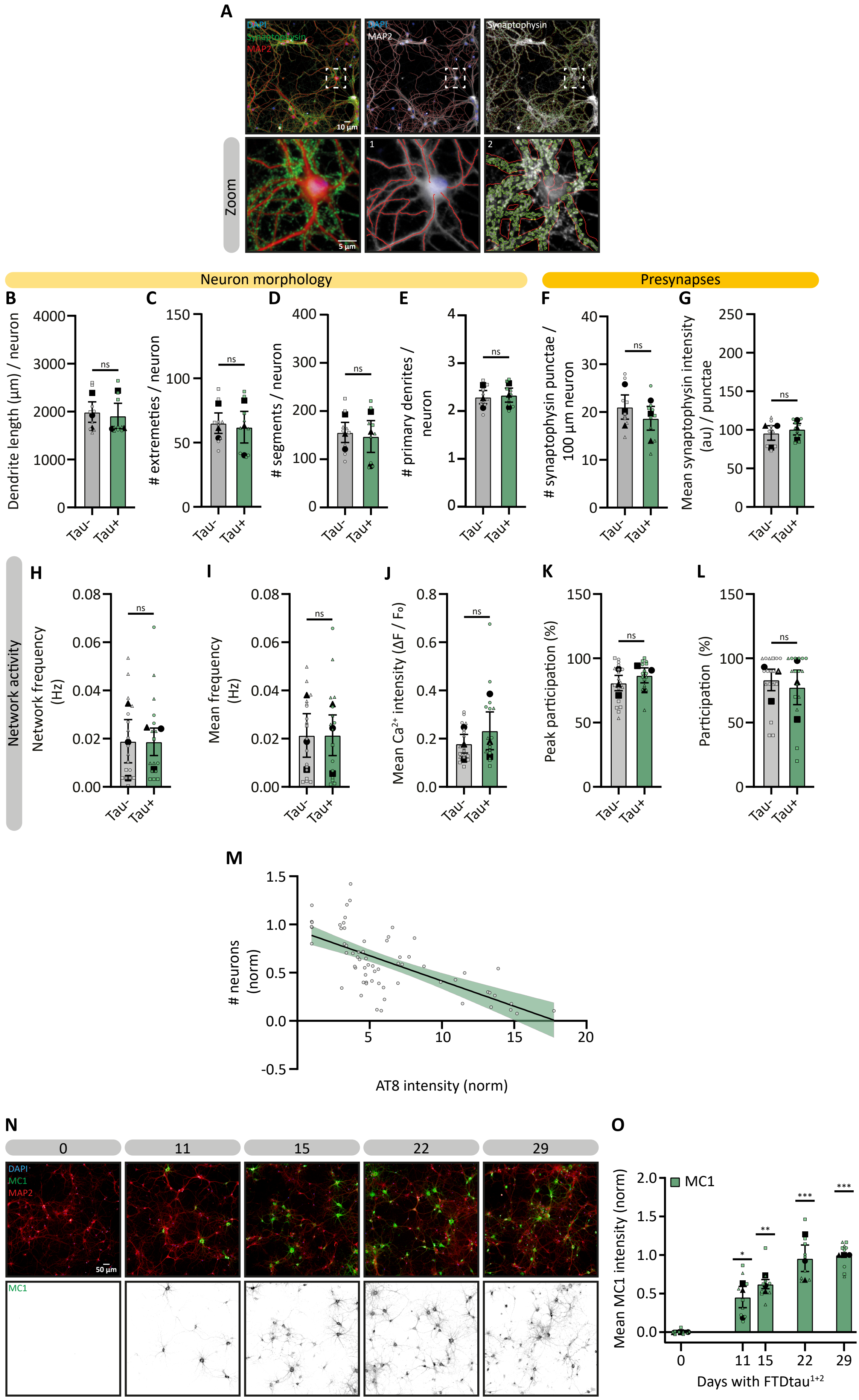
A
DAPI
Synaptophysin
MAP2
10 µm
Zoom
5 µm
Neuron morphology
Presynapses
B Dendrite length (µm) / neuron
C # extremeties / neuron
D # segments / neuron
E # primary dendrites / neuron
F # synaptophysin punctae / 100 µm neuron
G Mean synaptophysin intensity (au) / punctae
ns
Tau-
Tau+
Network activity
H Network frequency (Hz)
I Mean frequency (Hz)
J Mean Ca2+ intensity (ΔF / F0)
K Peak participation (%)
L Participation (%)
M
neurons (norm)
AT8 intensity (norm)
N
0
11
15
22
29
DAPI
MC1
MAP2
50 µm
O
MC1
Mean MC1 intensity (norm)
Days with FTDtau1+2

Fig. S1: Tau aggregation does not alter neuronal morphology, viability and network activity. A Representative high-content microscopy images showing the workflow for assessing neuron and pre-synapse morphology based on MAP2 (red, grey) and synaptophysin (green, grey) at day 15. Dashed squares indicate zoomed regions The detection of dendrites is based on the MAP2 signal (red traces, 1) and pre-synapses (yellow outline, 2) are defined using synaptophysin punctae within the dendrite region (red outline, 2). **B-G** Neuron and pre-synapse morphology in tau- and tau+ neurons at day 15. Dendrite length (**B**), number of extremities (**C**), number of segments (**D**) and number of primary dendrites (**E**) were normalised to neuron number. Number of pre-synapses (**F**) was normalised to dendrite length and the intensity of synaptophysin was measured within single pre-synapses (**G**) (N=3; n=9). **H-L**: Network activity in tau- and tau+ neuronal networks using calcium imaging analysed at day 15 including network frequency (**H**), mean frequency (**I**), mean calcium intensity (**J**), peak participation (**K**) and participation (**L**). Single datapoints represent recordings from 10 neurons per field of view (N=3; n=16-18). **M** Correlation between normalised AT8 intensity and neuron number after transduction with increasing concentrations of FTDtau^{1+2} ($y = -0.05x + 0.94$, $R^2 = 0.43$) analysed at day 15. **N** Representative high-content microscopy images at different FTDtau^{1+2} exposure durations as indicated showing MC1 (green) and MAP2 (red). MC1 channel is shown in greyscale. **O** Mean MC1 intensity normalised to 29 days FTDtau^{1+2} exposure (1) and untransduced (0) (N=3; n=9).

Nuclei are in blue (**A, N**). Data are presented as mean ± SEM. Nested t-test (**B-L**) or nested one-way ANOVA followed by Dunnett's post-hoc test (**O**) were used. *$P < 0.05$, **$P < 0.01$, ***$P < 0.001$, ns: not significant. Details of replicates and number of neurons analysed in table S1 and S2.

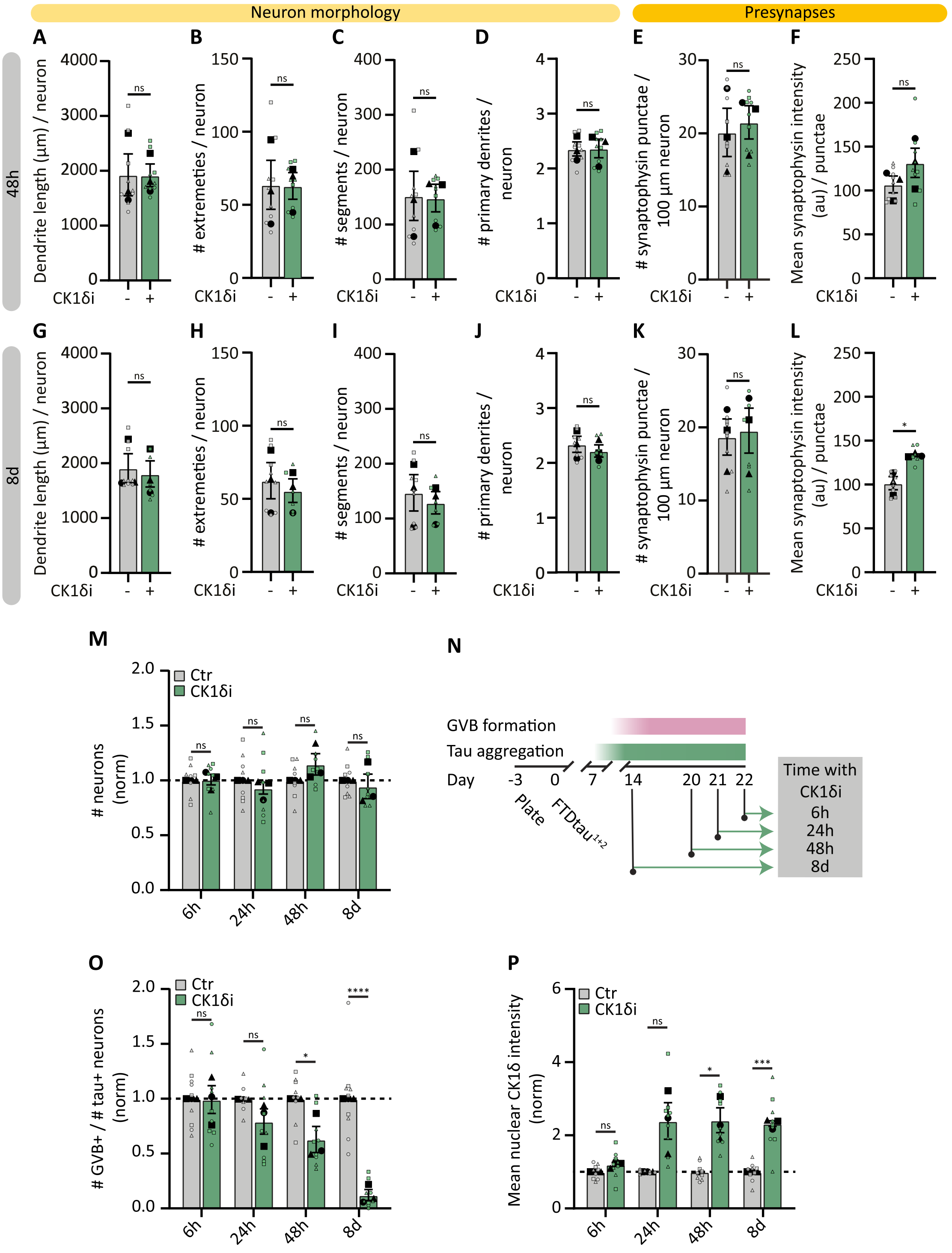

Neuron morphology
Presynapses
A
B
C
D
E
F
G
H
I
J
K
L
48h
8d
Dendrite length (µm) / neuron
extremeties / neuron
segments / neuron
primary denrites / neuron
synaptophysin punctae / 100 µm neuron
Mean synaptophysin intensity (au) / punctae
CK1δi
ns
*
M
N
O
P
Ctr
CK1δi
neurons (norm)
6h
24h
48h
8d
GVB formation
Tau aggregation
Day
-3
0
7
14
20
21
22
Plate
FTDtau1+2
Time with CK1δi
6h
24h
48h
8d
GVB+ / # tau+ neurons (norm)
****
Mean nuclear CK1δ intensity (norm)
***

Fig. S2: CK1δ inhibition does not alter neuronal morphology. A-L Neuron and pre-synapse morphology of tau+ neurons treated w/wo CK1δ_i for 48h or 8d analysed at day 15. Dendrite length (**A, G**), number of extremities (**B, H**), number of segments (**C, I**) and number of primary dendrites (**D, J**) were normalised to number of neurons. Number of pre-synapses (**E, K**) was normalised to dendrite length and the intensity of synaptophysin was measured within single pre-synapses (**F, L**) (N=3; n=6-9). **M** Tau+ neuron number upon CK1δ_i treatment for 6h, 24h, 48h or 8d analysed at day 15 normalised to untreated per timepoint (N=3; n=7-8). **N** Experimental timeline. **O, P** Fraction of GVB+/tau+ (pPERK based) neurons (**O**) and mean nuclear CK1δ intensity (**P**) upon CK1δ_i treatment analysed at day 22 normalised to untreated per timepoint (N=3; n=9).

Data are presented as mean ± SEM. Nested t-test was used (**A-L, M, O, P**). *$P < 0.05$, ***$P < 0.001$, ****$P < 0.0001$, ns: not significant. Details of replicates and number of neurons analysed in table S1 and S2.

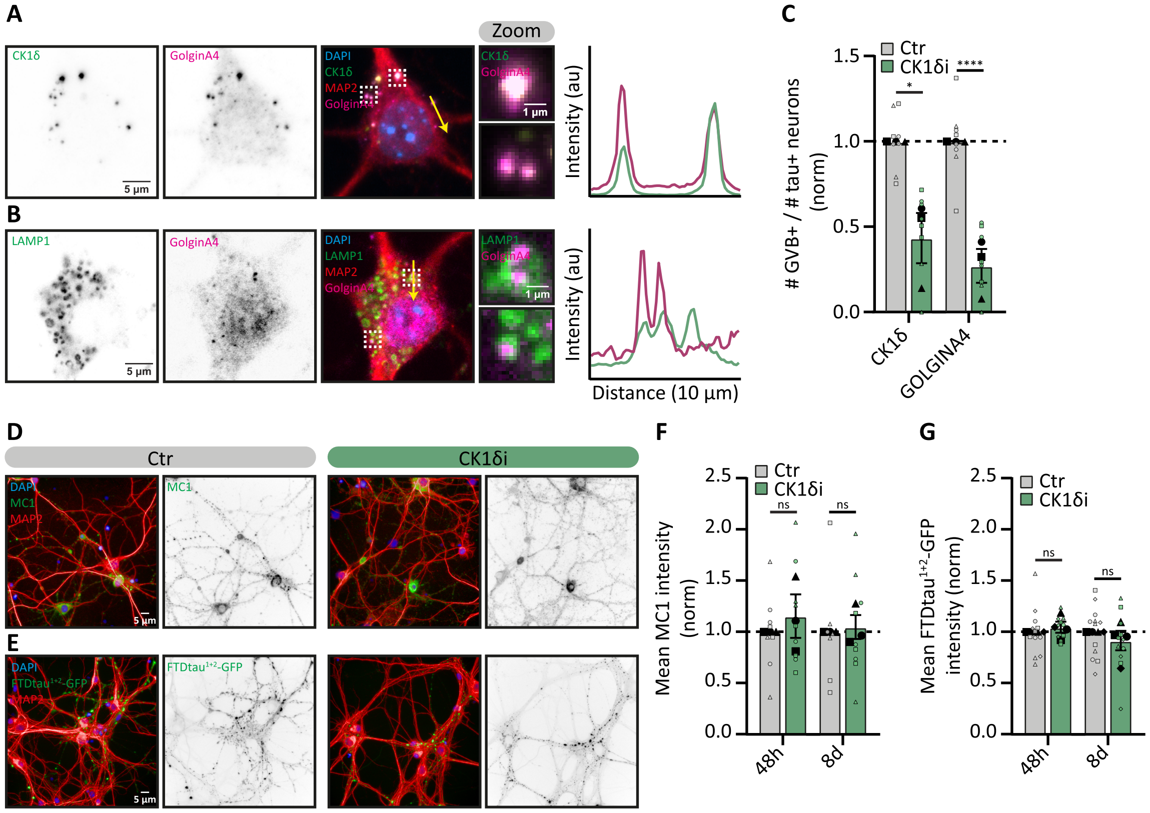
A
B
C
D
E
F
G
Zoom
CK1δ
GolginA4
DAPI
MAP2
LAMP1
5 µm
1 µm
Intensity (au)
Distance (10 µm)
Ctr
CK1δi
GVB+ / # tau+ neurons (norm)
GOLGINA4
*
****
MC1
FTDtau1+2-GFP
Mean MC1 intensity (norm)
Mean FTDtau1+2-GFP intensity (norm)
ns
48h
8d

Fig. S3: CK1δ inhibition does not affect GVB detection or tau pathology. A, B Representative confocal images of tau+/GVB+ neurons at day 22, showing CK1δ (green) and GOLGINA4 (magenta) (**A**), LAMP1 (green) and GOLGINA4 (magenta) (**B**) and MAP2 (red). Dashed squares indicate zoomed regions, yellow arrows indicate the location of the intensity profiles. **C** Fraction of CK1δ+ or GOLGINA4+ GVB+/tau+ neurons upon 48h of CK1δ_i treatment analysed at day 22 normalised to untreated per GVB-marker (N=3; n=8). **D, E** Representative high-content microscopy images of FTDtau^{1+2}- (**D**) or FTDtau^{1+2}-GFP- (**E**) transduced neurons treated w/wo CK1δ_i for 48h and analysed at day 15. Shown are MC1 (**D**) or FTDtau^{1+2}-GFP (green) (**E**) and MAP2 (red). **F-G** Mean MC1 (**F**) or FTDtau^{1+2}-GFP (**G**) intensity upon CK1δ_i for 48h or 8d normalised to untreated control (N=3-4; n=7-12).
Nuclei are in blue, separate channels are shown in greyscale (**A, B, D, E**). Data are presented as mean ± SEM. Nested t-test was used (**C, F, G**). *P < 0.05, ****P < 0.0001, ns: not significant. Details of replicates and number of neurons analysed in table S1 and S2.

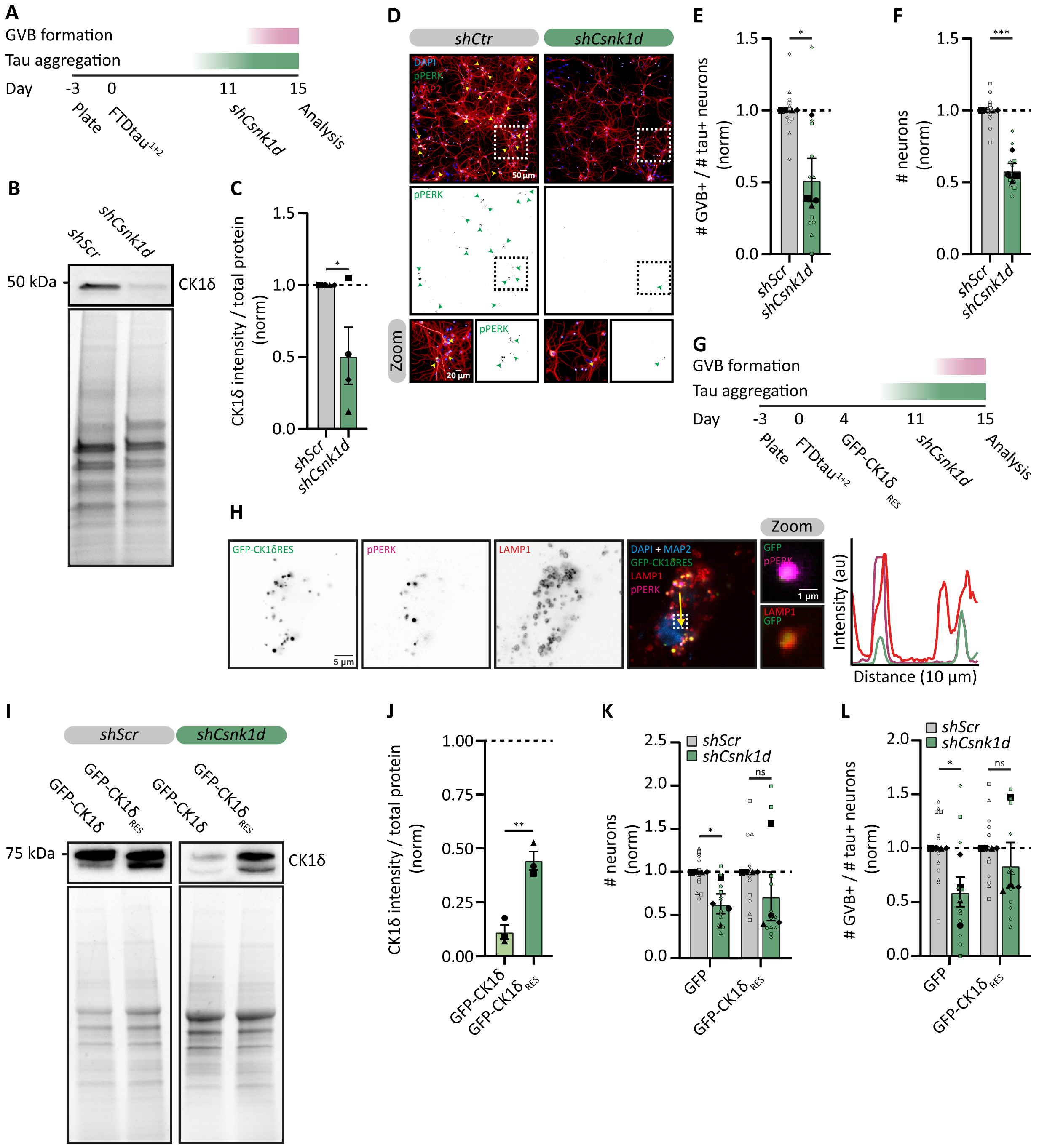
A
GVB formation
Tau aggregation
Day -3 0 11 15
Plate
FTDtau1+2
shCsnk1d
Analysis
B
shScr
shCsnk1d
50 kDa
CK1δ
C
CK1δ intensity / total protein (norm)
shScr
shCsnk1d
D
shCtr
shCsnk1d
DAPI
pPERK
MAP2
50 µm
pPERK
Zoom
20 µm
E
GVB+ / # tau+ neurons (norm)
shScr
shCsnk1d
F
neurons (norm)
shScr
shCsnk1d
G
GVB formation
Tau aggregation
Day -3 0 4 11 15
Plate
FTDtau1+2
GFP-CK1δ RES
shCsnk1d
Analysis
H
GFP-CK1δRES
pPERK
LAMP1
DAPI + MAP2
GFP-CK1δRES
LAMP1
pPERK
5 µm
Zoom
GFP
pPERK
1 µm
LAMP1
GFP
Intensity (au)
Distance (10 µm)
I
shScr
shCsnk1d
GFP-CK1δ
GFP-CK1δ RES
75 kDa
CK1δ
J
CK1δ intensity / total protein (norm)
GFP-CK1δ
GFP-CK1δ RES
K
shScr
shCsnk1d
neurons (norm)
ns
GFP
GFP-CK1δ RES
L
shScr
shCsnk1d
GVB+ / # tau+ neurons (norm)
ns
GFP
GFP-CK1δ RES

Fig. S4: CK1δ knock-down reduces GVB formation. A-F Tau+ neurons were transduced with *shScr* or *shCsnk1d* 4d before analysis at day 15. **A** Experimental timeline. **B** Representative western blot of CK1δ upon *Csnk1d* knock-down (KD). **C** CK1δ intensity over total protein upon *Csnk1d* KD normalised to *shScr* (N=4). **D** Representative high-content microscopy images of tau+ neurons transduced with *shScr* or *shCsnk1d* showing pPERK (green) and MAP2 (red). **E, F** Fraction of GVB+/tau+ (pPERK based) neurons (**E**) or tau+ neuron number (**F**) upon *Csnk1d* KD normalised to *shScr* (N=4; n=11-12). **G** Experimental timeline. **H** Representative confocal images of tau+/GVB+ neurons transduced with GFP-CK1δ_{RES} analysed at day 22. Shown are GFP (green), pPERK (magenta), LAMP1 (red) and MAP2 (blue). **I-L** Tau+ neurons were transduced with GFP-CK1δ or GFP-CK1δ_{RES} and *shScr* or *shCsnk1d* 11d and 4d, respectively, before analysis at day 15. **I** Representative western blot of CK1δ of neurons transduced with GFP-CK1δ or GFP-CK1δ_{RES} prior to *Csnk1d* KD. **J** CK1δ intensity over total protein upon GFP-CK1δ or GFP-CK1δ_{RES} expression prior to *Csnk1d* KD normalised to *shScr* per group. GFP-CK1δ_{RES} shows a partial rescue of CK1δ protein levels (N=3). **K, L** Number of GFP+ neurons (**K**) and the fraction of GVB+/tau+ (pPERK based) neurons (**L**) transduced with GFP or GFP-CK1δ_{RES} prior to *Csnk1d* KD normalised to *shScr* per group (N=4; n=12).

Nuclei are in blue, separate channels in greyscale, dashed squares indicate zoomed regions (**D, H**), arrowheads indicate GVB+ neurons (**D**), the yellow arrows indicates the location of the intensity profile (**H**). Data are presented as mean ± SEM. Unpaired t-test (**C, J**) and a nested t-test (**E, F, K, L**) were used. *P < 0.05, **P < 0.01, ***P < 0.001, ns: not significant. Details of replicates and number of neurons analysed in table S1 and S2.

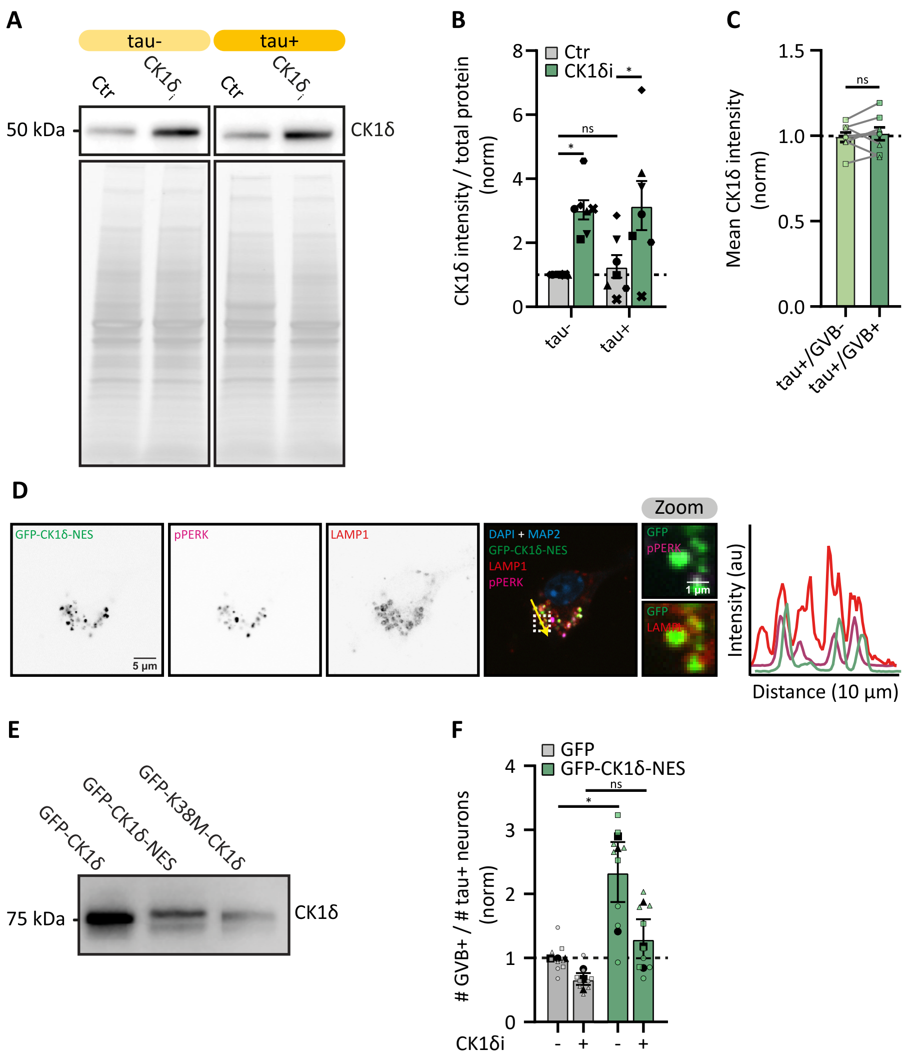
A
tau-
tau+
Ctr
CK1δi
50 kDa
CK1δ
B
CK1δ intensity / total protein (norm)
Ctr
CK1δi
ns
tau-
tau+
C
Mean CK1δ intensity (norm)
ns
tau+/GVB-
tau+/GVB+
D
GFP-CK1δ-NES
pPERK
LAMP1
DAPI + MAP2
Zoom
GFP
5 µm
1 µm
Intensity (au)
Distance (10 µm)
E
GFP-CK1δ
GFP-CK1δ-NES
GFP-K38M-CK1δ
75 kDa
CK1δ
F
GVB+ / # tau+ neurons (norm)
GFP
GFP-CK1δ-NES
ns
CK1δi

Fig. S5: Tau aggregation does not increase CK1δ levels. A Representative western blot of CK1δ of tau- or tau+ neurons treated w/wo CK1$δ_i$ for 48h analysed at day 15. **B** CK1δ intensity of tau- or tau+ neurons treated w/wo CK1$δ_i$ for 48h analysed at day 15 (N=7). **C** Mean somatic CK1δ intensity in tau+/GVB- and tau+/GVB+ (pPERK based) neurons, excluding the GVB-area, normalised to tau+/GVB- neurons analysed at day 15 (N=3; n=8). **D** Representative confocal images of tau+/GVB+ neurons transduced with GFP-CK1δ-NES at day 22. Shown are GFP (green), pPERK (magenta), LAMP1 (red) and MAP2 (blue). Nuclei are in blue, separate channels are shown in greyscale. A dashed square indicates the zoomed region, the yellow arrow indicates the location of the intensity profile. **E** Representative western blot of CK1δ of neurons transduced with GFP-CK1δ, GFP-CK1δ-NES or GFP-CK1δ-K38M and analysed at day 15. **F** Fraction of GFP+ GVB+/tau+ (pPERK based) neurons transduced with GFP or GFP-CK1δ-NES, treated w/wo 48h of CK1$δ_i$ analysed at day 22 and normalised to untreated GFP-transduced neurons (N=3; n=9).

Data are presented as mean ± SEM. One-way ANOVA followed by a Sidak's post-hoc test (**B**), paired t-test (**C**) and a nested one-way ANOVA followed by a Sidak's post-hoc test (**F**) were used. **P* < 0.05, *** *P* < 0.01, ns: not significant. Details of replicates and number of neurons analysed in table S1 and S2.

A

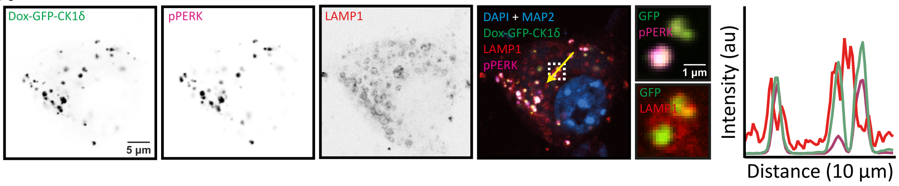
Dox-GFP-CK1δ
5 µm
pPERK
LAMP1
DAPI + MAP2
Dox-GFP-CK1δ
LAMP1
pPERK
GFP
pPERK
1 µm
GFP
LAMP1
Intensity (au)
Distance (10 µm)

B

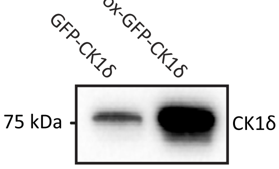
GFP-CK1δ
Dox-GFP-CK1δ
75 kDa
CK1δ

C

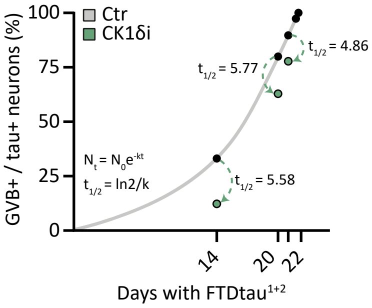
GVB+ / tau+ neurons (%)
100
75
50
25
0
Ctr
CK1δi
t1/2 = 5.77
t1/2 = 4.86
t1/2 = 5.58
Nt = N0e-kt
t1/2 = ln2/k
14
20
22
Days with FTDtau1+2

Fig. S6: GVBs are stable structures. A Representative confocal images of tau+/GVB+ neurons transduced with Dox-GFP-CK1δ and treated with Dox for 48h analysed at day 22. Shown are GFP (green), pPERK (magenta), LAMP1 (red) and MAP2 (blue). Nuclei are in blue, separate channels are shown in greyscale. A dashed square indicates the zoomed region, the yellow arrow indicates the location of the intensity profile. **B** Representative western blot of CK1δ of neurons transduced with Dox-GFP-CK1δ and treated with Dox for 48h analysed at day 15. **C** Visualisation of GVB half-life ($t_{1/2}$) based on the polynomial equation obtained from Fig. 1H ($y = 0.05x^2 - 0.3x + 0.2$) to calculate the expected amount of GVB+ neurons after different exposure periods to tau aggregation (grey line) and the observed amount of GVB+ neurons upon 24h, 48h and 8d of CK1δ_i (fig. S2O). Assuming an exponential decay ($N_t=N_0e^{-kt}$ and $t_{1/2}=\ln2/k$), $t_{1/2}$ was calculated using the ratio of expected over observed amount of GVB+ neurons upon CK1δ_i. The dotted green arrows indicate the difference in expected and observed amount of GVB+ neurons per timepoint.

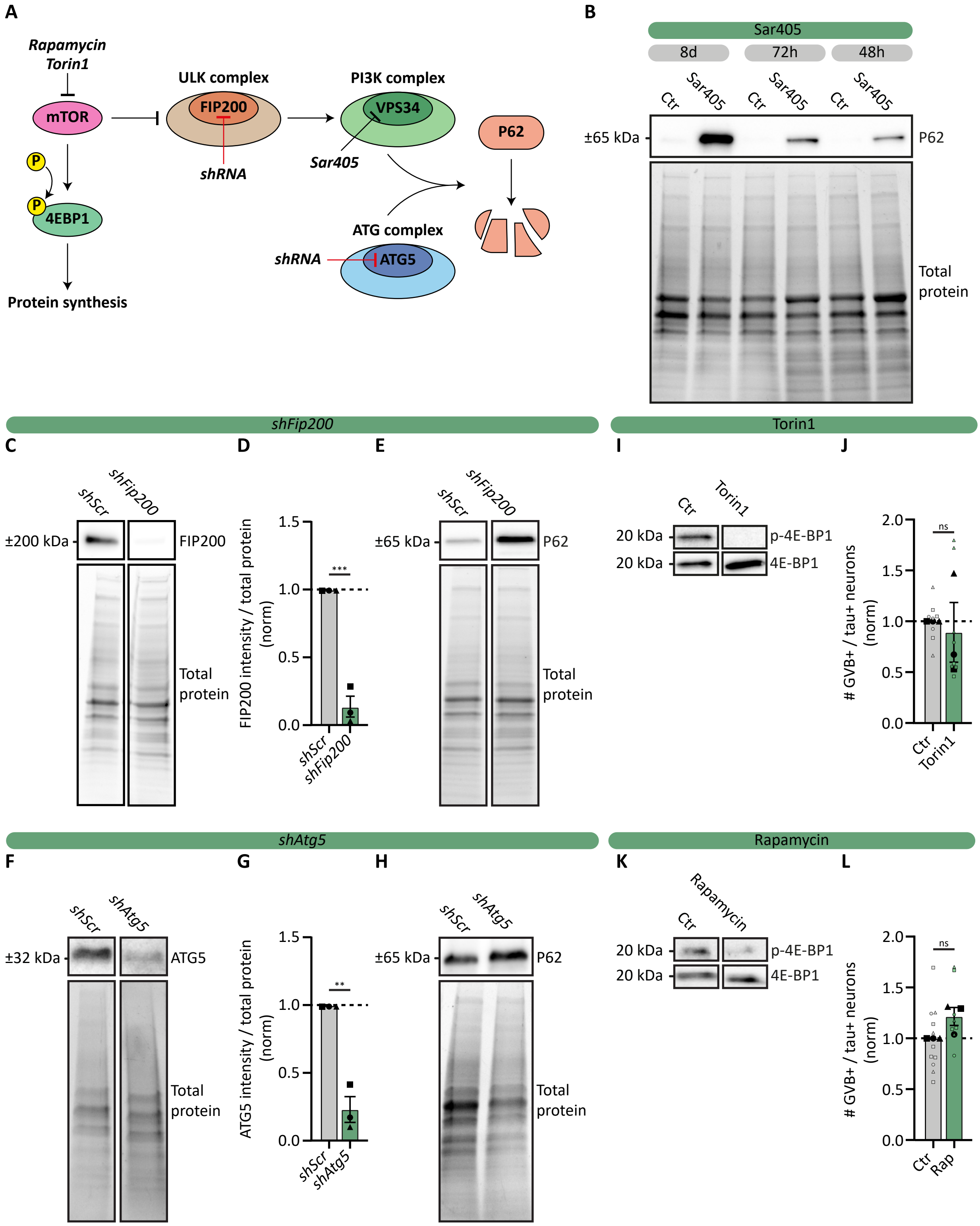
A
Rapamycin
Torin1
mTOR
P
4EBP1
Protein synthesis
ULK complex
FIP200
shRNA
PI3K complex
VPS34
Sar405
ATG complex
ATG5
P62
B
Sar405
8d
72h
48h
Ctr
Sar405
±65 kDa
P62
Total protein
shFip200
C
shScr
shFip200
±200 kDa
FIP200
Total protein
D
FIP200 intensity / total protein (norm)
***
E
±65 kDa
P62
Torin1
I
Ctr
Torin1
20 kDa
p-4E-BP1
4E-BP1
J
GVB+ / tau+ neurons (norm)
ns
shAtg5
F
shScr
shAtg5
±32 kDa
ATG5
G
ATG5 intensity / total protein (norm)
**
H
Rapamycin
K
L
Ctr
Rap

Fig. S7: Validation of autophagy interventions. A Schematic of interventions used to block mTOR-dependent autophagy. **B** Representative western blot of P62 of neurons treated w/wo Sar405 for 8d, 72h or 48h analysed at day 15. **C** Representative western blot of FIP200 of neurons transduced with *shScr* or *shFip200* 8d analysed at day 15. **D** FIP200 intensity over total protein upon *Fip200* knock-down (KD) normalised to *shScr* (N=3). **E** Representative western blot of P62 of neurons transduced with *shScr* or *shFip200* 8d analysed at day 15. **F** Representative western blot of ATG5 of neurons transduced with *shScr* or *shAtg5* 8d analysed at day 15. **G** ATG5 intensity over total protein upon *Atg5* KD normalised to *shScr* (N=3). **H** Representative western blot of P62 of neurons transduced with *shScr* or *shAtg5* 8d analysed at day 15. **I, K** Representative western blots of p-4E-BP1 and 4E-BP1 of neurons treated w/wo Torin1 (**I**) or rapamycin (**K**) for 48h analysed at day 15. **J, L** Fraction of GVB+/tau+ (pPERK (**J**) or CK1δ (**L**) based) neurons upon 48h of Torin1 (**J**) or rapamycin (**L**) treatment normalised to untreated per timepoint analysed at day 15 (N=3; n=8-11).
Data are presented as mean ± SEM. Unpaired t-test (**D, G**) and nested t-test (**J, L**) were used **P < 0.01, ***P < 0.001, ns: not significant. Details of replicates and number of neurons analysed in table S1 and S2.

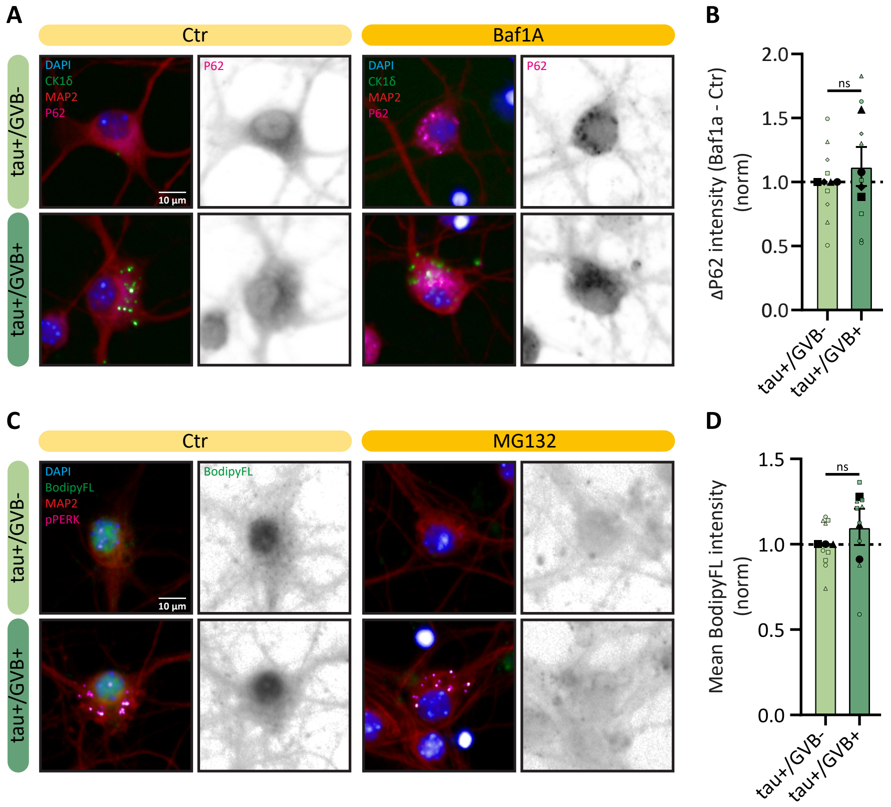

A
Ctr
Baf1A
tau+/GVB-
tau+/GVB+
DAPI
CK1δ
MAP2
P62
10 µm
B
ΔP62 intensity (Baf1a - Ctr) (norm)
ns
2.0
1.5
1.0
0.5
0.0
tau+/GVB-
tau+/GVB+
C
Ctr
MG132
DAPI
BodipyFL
MAP2
pPERK
10 µm
D
Mean BodipyFL intensity (norm)
ns
1.5
1.0
0.5
0.0

Fig. S8: Autophagic flux and proteasomal activity levels are not different between tau+/GVB- and tau+/GVB+ neurons. A Representative high-content microscopy images of tau+/GVB- and tau+/GVB+ neurons treated w/wo BafA1 for 4h analysed at day 15. Shown are CK1δ (green), MAP2 (red) and P62 (magenta). **B** Difference in mean somatic P62 intensity between BafA1 and Ctr in both tau+/GVB- and tau+/GVB+ (CK1δ based), as a readout for autophagic flux, normalised to tau+/GVB- neurons analysed at day 15 (N=4; n=8) **C** Representative high-content microscopy images of tau+/GVB- and tau+/GVB+ neurons treated w/wo MG132 for 1h prior to the addition of the proteasomal activity probe Me4BodipyFL-Ahx3Leu3VS analysed at day 15. Shown are the proteasomal activity probe (green), pPERK (magenta) and MAP2 (red). **D** Mean somatic proteasomal activity probe intensity in tau+/GVB- and tau+/GVB+ (pPERK based), background corrected for MG132 intensity per group and normalised to tau+/GVB- neurons analysed at day 15. (N=3; n=9). Nuclei are in blue, separate channels are shown in greyscale (**A, C**). Data are presented as mean ± SEM. A nested t-test was used (**B, D**). ns: not significant. Details of replicates and number of neurons analysed in table S1 and S2.

A

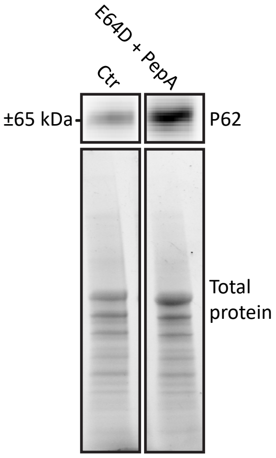

Fig. S9: Lysosomal inhibition using E64D and Pepstatin A reduces P62 degradation. A Representative western blot of P62 of neurons treated w/wo E64D and Pepstatin A (PepA) for 24h analysed at day 15.

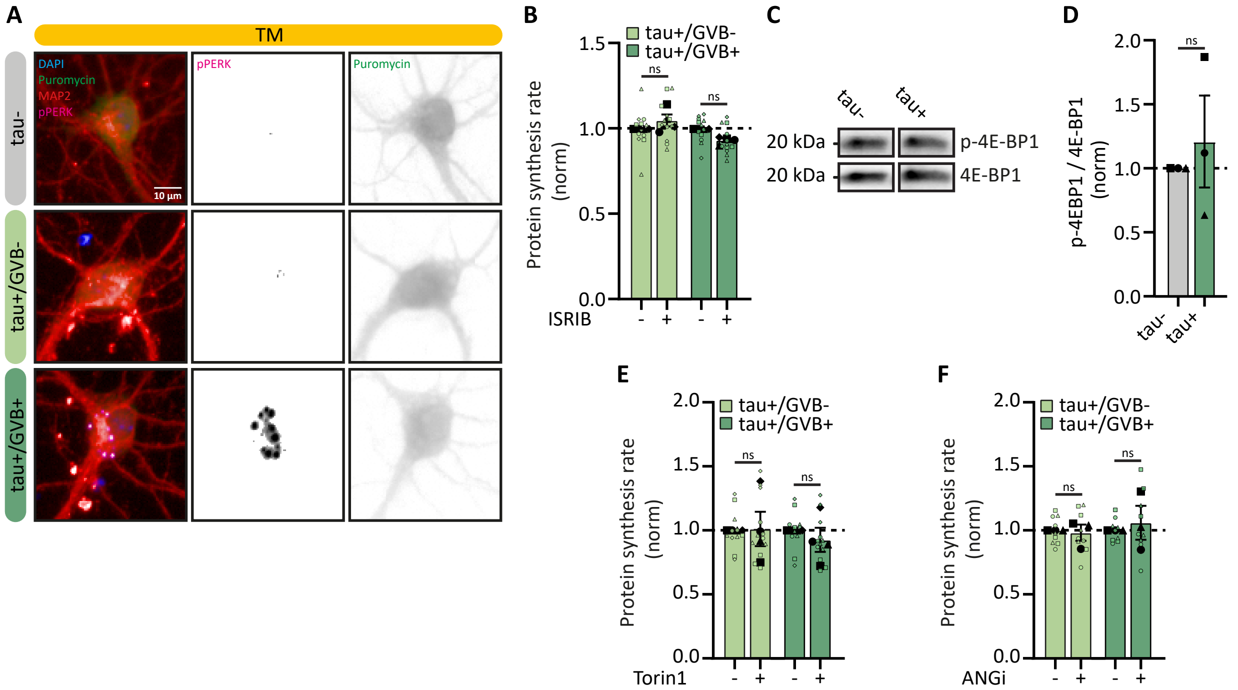
A
TM
tau-
tau+/GVB-
tau+/GVB+
DAPI
Puromycin
MAP2
pPERK
10 µm
pPERK
Puromycin
B
tau+/GVB-
tau+/GVB+
Protein synthesis rate (norm)
ns
ns
ISRIB
- + - +
C
tau-
tau+
20 kDa
p-4E-BP1
20 kDa
4E-BP1
D
p-4EBP1 / 4E-BP1 (norm)
ns
tau-
tau+
E
tau+/GVB-
tau+/GVB+
Protein synthesis rate (norm)
ns
ns
Torin1
- + - +
F
tau+/GVB-
tau+/GVB+
Protein synthesis rate (norm)
ns
ns
ANGi
- + - +

Fig. S10: GVB formation and regulation does not depend on transient stress pathways. **A** Representative high-content microscopy images of tau-, tau+/GVB- and tau+/GVB+ neurons treated with TM for 24h analysed at day 22 showing MAP2 (red), pPERK (magenta) and puromycin (green). Nuclei are in blue, separate channels are shown in greyscale. **B** Puromycin intensity was quantified to assess protein synthesis rate in tau+/GVB- and tau+/GVB+ (pPERK based) neurons treated w/wo ISRIB for 2h analysed at day 22, normalised to untreated per group (N=3; n=9). **C** Representative western blots of p-4E-BP1 and 4E-BP1 of tau- and tau+ neurons analysed at day 15. **D** p-4E-BP1 intensity over 4E-BP1 intensity normalised to tau- analysed at day 15 (N=3). **E, F** Puromycin intensity was quantified to assess protein synthesis rate in tau+/GVB- and tau+/GVB+ (pPERK based) neurons treated w/wo Torin1 (**E**) or angiogenin inhibitor (ANG_i, **F**) for 24h analysed at day 15, normalised to untreated per group (N=3; n=9).
Data are presented as mean ± SEM. Nested t-test (**B, E, F**) and unpaired t-test (**D**) were used. ns: not significant. Details of replicates and number of neurons analysed in table S1 and S2.

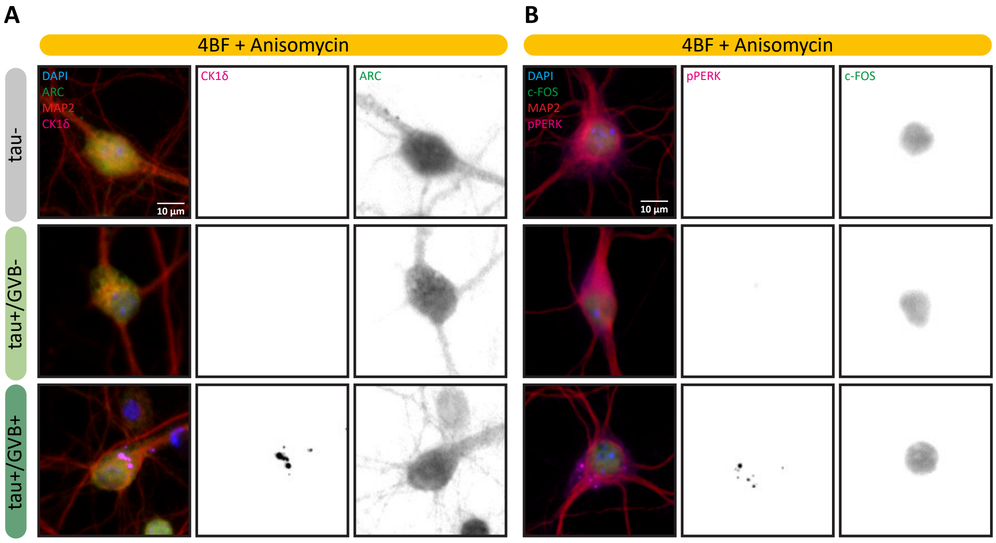
A
B
4BF + Anisomycin
4BF + Anisomycin
tau-
tau+/GVB-
tau+/GVB+
DAPI
ARC
MAP2
CK1δ
CK1δ
ARC
10 µm
DAPI
c-FOS
MAP2
pPERK
pPERK
c-FOS
10 µm

Fig. S11: LTP-dependent induction of immediately early genes (IEGs) is dependent on protein synthesis. A, B Representative high-content microscopy images of tau-, tau+/GVB- and tau+/GVB+ neurons treated with Ani prior to treatment with 4BF for 4h. Shown are ARC (**A**) or c-FOS (**B**) (green), MAP2 (red) and CK1δ (magenta) (**A**) or pPERK (magenta) (**B**). Nuclei are in blue, separate channels are shown in greyscale (**A, B**).

Table S1: Overview of all technical replicates. Table summarising experimental details of biological (N) and technical (n) replicates and number of neurons analysed for all datasets in this study.

Table S2: Overview of the raw data. Table containing all the absolute values used to create the corresponding graphs, including the number of tau-, tau+, tau+/GVB- and tau+/GVB+ neurons analysed, the percentage of tau+/GVB+ neurons per well, the intensity measurements and other neuronal parameters.

Table S3: Data table of proteomics dataset.

Video S1: GVBs are stable, dynamic structures. Representative live imaging of a tau+ neuron transduced with GFP-CK1δ (grey) and imaged at day 21. An image was taken every 20 min for the duration of 9.20h. Although GVBs very rarely appeared or disappeared, this example shows a GVB disappearing (red arrow) and one appearing (black arrow).
